# National Sentiments and Regional Flavour- A Socio-economic Study of Huvina Hadagali Jasmine

**DOI:** 10.12688/f1000research.153101.1

**Published:** 2024-09-24

**Authors:** Jyeshtaraja Joisa, Harisha G Joshi, Kavitha T C, Javed Bhasha

**Affiliations:** 1 Department of Commerce, Manipal Academy of Higher Education, Manipal, Karnataka, 576104, India; 2 Department of Commerce, GANGAVATHI BHAGYAMMA RURAL DEGREE COLLEGE, HUVINHADAGALI, Huvina Hadagali, Karnataka, 583219, India

**Keywords:** Hadagali Jasmine, Socio-economic study, 2-Step cluster analysis, Floriculture, Jasmine flower, floriculture market.

## Abstract

**Background:**

This socio-economic analysis studies the influence of jasmine production on the economic well-being of farmers in Huvina Hadagali, a region known for its high-quality jasmine flowers. The Vijaya Nagara district’s Havina Hadagali area is well known throughout the country for its jasmine flower farming. In addition to being referred to as Mallige Nadu, this location is also known as Malligeya Tavaru. The cultivation of the jasmine flower is protected by the Geographical Indication (GI) Tag, and this flower has been popular in this region for a substantial amount of time.

**Methods:**

Data was collected from a sample of 364 jasmine growers using a structured questionnaire in Huvina Hadagali, Vijayanagar district. The data focused on different socio-economic factors such as income levels, employment, market access, and agricultural techniques. The study is analysed using IBM SPSS through frequency analysis and 2-step clustering.

**Result:**

The results demonstrate that the cultivation of jasmine makes a substantial contribution to the local economy, serving as a main or additional source of income for numerous households. Jasmine farming often contributes 40% of the whole household income, and during peak seasons, it provides significant economic advantages. Nevertheless, the highlighted obstacles were volatile market pricing, pest infestations, and limited access to contemporary farming practices. The study emphasises the crucial significance of cooperative societies and local marketplaces in stabilising income and offering essential resources and training to farmers.

**Conclusion:**

The research highlights the necessity of governmental interventions focused on developing market infrastructure, offering financial assistance, and improving access to agricultural innovations to maintain and augment the economic advantages of jasmine cultivation in Huvina Hadagali.

## 1. Introduction

India has a long-standing association with flowers, as shown in their utilisation in ancient texts, traditions, and paintings ( Bhattacharya, 2014). The Rigveda, an old sacred literature, acknowledges the significance of flowers in ceremonies and everyday life. Flowers are seen as having several uses. Flowers’ spiritual and aesthetic importance is emphasised in the Rigveda ( Balkrishna et al., 2019). Flowers have an essential role in both ceremonies and in day-to-day living. The development of floriculture in India has been significantly influenced by several dynasties and civilisations throughout its history. These dynasties and cultures have improved floriculture by introducing new species and innovative cultivation methods ( P.Renganathan & Dr. A. Gopalakrishnan, 2019). The large terrain of India, which is also rich in cultural legacy and floral practices, yields a diverse range of flowers planted across the country. Patel et al. (2022) highlight that jasmine’s captivating fragrance and cultural significance set it apart from other popular flowers. In Indian tradition, the jasmine is revered as a sign of purity, grace, and welcome. As a subfield of horticulture, floriculture is a specialised field that focuses on cultivating attractive blooming plants ( Dr. R. Pichumani & Latha R, 2018). These plants are grown for commercial use, such as selling them or as raw materials for the cosmetics industry. There has been a constant increase in the demand for floricultural products in both the home and international markets ( Ganga et al., 2019). Cut flowers, which have great potential for worldwide trade, result from India’s significant progress in flower agriculture ( Carton & Parigot, 2022).

Farming flowers and decorative plants in India addresses various activities, including cultivation, processing, retailing, and exporting these products. India is an ideal floriculture destination throughout its regions because of its varied agro-climatic zones, excellent environment, and abundant competent labour ( Swain & Maurya, 2021). The gorgeous white blossoms and entrancing smells that become more intense throughout the nighttime hours of this floral fragment, which belongs to the genus Jasminum, are the reasons for its great worth. It is believed that jasmine has been cultivated in India for a very long time, reaching back to ancient times. Allusions from classical works, poetry, and folklore Balkrishna et al. (2019) make this point abundantly clear. India is one of the world’s largest jasmine-growing countries; major cultivation areas are found in parts of Maharashtra, Uttar Pradesh, West Bengal, and the Southern States of Tamil Nadu, Karnataka, Andra Pradesh, and Kerala ( Yoganandan, 2020). It is possible to cultivate jasmine in an excellent environment thanks to favourable climatic conditions, which include high temperatures, moderate precipitation, and soil that drains well. According to Palanisingh et al. (2022), Karnataka, situated in southern India, is delighted because it cultivates various colours and flowers. This flourishing sector is deeply rooted in the state’s cultural heritage and can be attributed to the ideal agricultural and climatic circumstances. In this detailed inquiry, the various landscapes of floriculture in the state of Karnataka are investigated. Particular attention is paid to the growing methods utilised and Jasmine’s economic worth and cultural significance. Karnataka has emerged as a significant centre for floriculture in India, with a flourishing industry that includes the whole process, from cultivating to selling cut flowers and decorative plants ( Yakandawala, 2016).

The commercial cultivation of jasmine in eastern India serves both the domestic and international markets, with a significant portion of the production consumed locally for religious and cultural purposes ( Landran & Gupta, 2017). Jasmine is the flower of the jasmine flower group. According to Cnaan et al. (2014), the jasmine flower market is dynamic and influenced by several factors, such as festivals, weddings, seasonal demand, and religious occasions. While traditional channels like neighbourhood flower markets, houses of worship, and florists remain essential sales channels, e-commerce has opened new opportunities for speaking with customers directly ( D’souza & Joshi, 2020). India’s agricultural economy has significantly benefited from the proliferation of jasmine, which has had a substantial economic impact. It is profitable for small-scale or economically disadvantaged farmers, in particular, who rely on jasmine production as their primary source of income. The Vijaya Nagara district’s Havina Hadagali area is well known throughout the country for its jasmine flower farming. In addition to being referred to as Mallige Nadu, this location is also known as Malligeya Tavaru. The cultivation of the jasmine flower is protected by the Geographical Indication (GI) Tag, and this flower has been popular in this region for a substantial amount of time. It was common practice during the time of the Vijayanagara Empire to transport jasmine flowers from the Tungabhadra River to the temple of Lord Virupaksa in Hampi daily by boat ( Nagaveni, 2018). To determine the socio-economic standing of the jasmine producers, most farmers are seen to be engaged in the cultivation of G.I.-tagged jasmine. It is essential to investigate the history of Hadagali Jasmine and understand how the growers feel about adding value and production.

## 2. Literature review

Floriculture is a subfield of horticulture that focuses on cultivating, marketing, and aesthetic arrangement of flowers and decorative plants for commercial purposes ( Krishnaveni & Pethalakshmi, 2017). This includes the cultivation of annuals, biennials, and perennials ( Anumala & Kumar, 2021). Plant-cut foliage, seed bulbs, tubers, rooted cuttings, or dried flowers or leaves primarily comprise blooms removed from the plant ( Beneragama & Peiris, 2016). The transition from traditional crops to high-value-added crops, such as horticulture and floriculture, is taking place gradually in South Asian countries that are economically developing ( Saripalle, 2016). India’s youth are benefiting from increased revenue, profits, and employment prospects thanks to the floriculture industry, which is also supporting the participation of women to a substantial degree and boosting exports ( Krishnaveni & Pethalakshmi, 2017). There have been notable breakthroughs in India’s flower cultivation, particularly in creating blooms with extensive export potential. This industry creates work opportunities and generates higher incomes for women ( Anumala & Kumar, 2021). In terms of floriculture, India has a long and illustrious history, which has resulted in a significant number of advantages. Agricultural products and trade are essential to India’s economy. It has been decades since India’s small and marginal farmers could lessen their pricing risk due to improved agricultural marketing ( Agrawal et al., 2022; Markelova et al., 2009). Flower is a commodity that has a concise shelf life. Marketing is a significant challenge. Rural areas are responsible for producing flowers, yet urban areas are the principal consumers of these flowers. There is a need for flowers in rural areas only for special events, such as weddings and festivals. It is, therefore, necessary for the growers to locate a market in metropolitan areas. Only a few types of flowers, including rose gladiolus, chrysanthemums, jasmine, and orchids, are produced and exported from India ( Lepcha et al., 2020).

One of the most essential ornamentals and flavourful plants, jasmine, has been traditionally cultivated in several different countries. Producing essential oils as “concrete” and “absolute” is another application. These oils are utilised in the cosmetic and perfume industries ( Ray et al., 2014). Fully open flowers contain the highest possible aroma and must be harvested to extract concrete. Since ancient times, small farmers have traditionally sold their crops to intermediaries at the farm gate, frequently at a low price ( Gills et al., 2017). As a result of their heavy reliance on wholesale brokers and traders for market knowledge and credit facilities, most small and medium farmers in these Nations are bound into informal contractual arrangements. A manual classification system is used to determine the quality of the flowers ( Krishnaveni & Pethalakshmi, 2017). The freshness of the flowers is diminished during this process, which takes a significant amount of time. As a result, judging the quality of their products has become extremely difficult. Considering that these tasks are carried out manually and require significant labour, it is essential to automate determining the quality of flowers ( Thakur et al., 2023).

Agricultural producers worldwide, including those in India, face significant challenges from market-related constraints such as agricultural produce’s marketing and supply chain ( Yapa, 2022). Over the past few years, one of the most critical concerns raised in India is the effectiveness of the marketing of agricultural products. Additionally, a smaller proportion of consumer rupees is reaching growers, which is regarded to be the fundamental reason for high and volatile consumer prices. Inadequate marketing infrastructure and poor effectiveness of marketing channels are also believed to be related to this issue ( Sathish & Rajamohan, 2019). During COVID-19, there was a shortage of labourers because of restrictions placed on migrant workers. Although the consumer’s purchase rate is high, the number of agricultural commodities that farmers produce is low ( Sandeep Kumar et al., 2020). In the context of a changing climate and diminishing land resources, water scarcity, pests, and illnesses, an ever-increasing population, low productivity under open conditions, and changes in consumer choice are the causes that are driving more and more people to switch to protected cultivation ( Pachiyappan et al., 2022). When it comes to the adoption and implementation of erosion control measures, the issue of land tenure plays a significant role ( Sohal et al., 2023). This is especially true for technologies that require extensive planning and commitment over an extended period. Most farmers who grow flowers sell their produce to commission agents in the flower mandi without adding value. The economic well-being of flower producers needs to have post-harvest management and value-addition programmes in place within the floriculture industry. The introduction of novel marketing arrangements can potentially alter market relations in a manner that serves the interests of smallholders ( Gills et al., 2017).

Producer organisations are in an advantageous position to capitalise on these novel methods. In addition to displaying a great interest in agricultural technology, young people who are unemployed and educated but have no interest in traditional agriculture also exhibit a strong interest in developing agricultural technologies ( Thakur et al., 2023). Additionally, it has been noted that the costs associated with the protected cultivation of floral crops were superior to the yield realised from the flowers ( Pachiyappan et al., 2022). It is important to note that specific technology categories, such as enhanced varieties and chemical input, have a higher possibility of being adopted on more giant farms, raising questions about these technologies’ scalability. Nevertheless, it is pretty different from tradition to assess the degree to which farmers encounter credit constraints instead of merely determining their credit accessibility. This is even though the relevance of agricultural finance is obvious—the implementation of higher order. Compared to deploying a single technology, integrating multiple technologies results in more significant dividends for farmers regarding form yield and income ( Nsabimana & Adom, 2024). Various integrated technologies were investigated, and the study’s findings revealed that the technological mining involving crop and soil improvements had the most substantial influence. Crop intensification projects aim to achieve a sustainable improvement in productivity by acquiring improvised seeds, fertilisers, and pesticides to raise crop production potentials. With population expansion, climate change, rising demand for high-quality produce, dwindling band holdings, and more significant pressure on resources, there is a duty to employ modern crop production methods for protected cultivation ( Pachiyappan et al., 2022). This obligation is one of the reasons why protected cultivation is so important. Through cutting-edge structures, protected agriculture is a high-tech method that allows crop production in an environment controlled and protected from bad climatic conditions. Protected cultivation is more sustainable than open cultivation methods as the impact of climate is reduced significantly because the environment is controlled. Additionally, the inputs, which include fertilisers, pesticides, and water, are utilised more effectively than in open cultivation methods. Furthermore, the increased productivity and improved quality of the produce ensures a higher return on investment. When it comes to protected cultivation, the implementation of contemporary technology like artificial intelligence, robotics for plucking, and other similar technologies, as well as the utilisation of the Internet of Things (IoT) or sensor-based irrigation scheduling, would contribute to an increase in both efficiency and income for farmers in the region. To support protected gardening more, the Goods and Services Tax (GST) on playhouses and warehouses in India must be lowered ( Anumala & Kumar, 2021). This should also be done for precision farming, value additions, and creating an integrated flower cold chain. It has been determined by the government of India that floriculture is a sunrise industry, and it has been given the status of being entirely export-focused. As a result of the consistent rise in the demand for flowers, floriculture has emerged as one of the most important commercial business sectors within the agricultural sector. Over the previous few decades, the topics of ecological tourism, emerging mode, and rural regeneration have been the focus of studies that have been conducted for a considerable amount of time. Agricultural practices that are based on ecological principles and emphasise environmental sustainability are referred to as ecological agriculture, also called eco-agricultural ( Fan et al., 2024).

The proliferation of governance mechanisms, such as agricultural contracts, farmer’s groups, estate farming, and farm non-governmental organisations, further drives the change by improving vertical integrational horizontal coordination and, as a result, making it easier for small farmers to access high-value markets because of this improvement ( Jagadeesh, 2020). High market value participation suits family income, productivity, and economic growth and development in rural areas. For this reason, developmental policymakers need to understand the patterns surrounding high-market-value participation ( Ola & Menapace, 2020). In addition to filling in coordination gaps, market access can be improved through collaboration, which can help correct some of the imperfections in the market, such as excessive transaction costs and the absence of credit markets ( Ola & Menapace, 2020). Furthermore, when farmers combine their financial and labour resources, they are better able to gather the necessary information, meet quality standards, and operate on a large scale. This allows them to sell their products to new local or foreign markets, which would otherwise be out of reach for smaller producers ( Shah et al., 2022).

## 3. Study design

### 3.2 Methods

In this research, the primary aim is to investigate various aspects related to Jasmine cultivation, focusing on cultivators in the Huvina Hadagali region. The study employs a questionnaire-based approach to gather data from Jasmine cultivators, with subsequent analysis utilising frequency and percentage analysis and two-step cluster analysis using IBM SPSS. A cross-sectional research design is adopted to collect data from Jasmine cultivators in the Huvina Hadagali region simultaneously. The study employs a purposive sampling technique to select participants actively involved in Jasmine cultivation in the Huvina Hadagali region. The sample size for this study is determined to be 364 Jasmine cultivators from Huvina Hadagali. A structured questionnaire is developed to collect data from the participants. The questionnaire consists of closed-ended questions covering various aspects related to Jasmine cultivation, including cultivation practices, challenges faced, yield, market access, and socio-demographic characteristics of the cultivators. Upon collecting the questionnaire responses, the data is subjected to frequency and percentage analysis to examine the distribution of responses across different variables. This analysis provides insights into the prevalence of various practices, challenges, and characteristics among Jasmine cultivators in Huvina Hadagali. Additionally, a two-step cluster analysis is conducted using IBM SPSS version 27 (Statistical Package for the Social Sciences) to identify distinct groups or clusters within the sample population based on their responses to the questionnaire. This analytical technique helps uncover patterns or relationships among the variables and allows for a deeper understanding of the heterogeneity within the population of Jasmine cultivators.

## 4. Findings and Discussion

### 4.1 Demographic analysis

Some exciting trends may be seen in the demographic analysis of the farmers in Huvina Hadagali. It demonstrates how young and active the farming community is, with a sizable percentage (36.8%) of its members being under 30 years old. In addition, among the farming community’s decently educated members, a sizeable percentage (39.6%) have completed higher education beyond the twelfth grade. The insights shed essential light on the changing dynamics within the Havina Hadagali agricultural community, emphasising how family structure, age, gender, and education affect the makeup and operations of the group. The increasing number of nuclear families and the three- to five-member majority in agricultural families are signs of the community’s adaptation and resilience.

Table 2 thoroughly examines Huvina Hadagali farmers’ economic conditions, concentrating on their sources of revenue and ways to finance their farming operations. A sizable portion of farmers—52.2%—use the production of jasmine as their primary source of yearly net income per acre, with the majority making between Rs 10,000 and Rs 15000. Interestingly, most farmers in Havina Hadagali (52.7%) use borrowed and personal funds to finance their agricultural operations. This emphasises how the farming sector needs to diversify financially to maintain viability.

**Table 1.** Demographic factors.

Demographics factor		
		Percentage %
Age	Up to 30 years	36.8
	31 – 40 years	27.5
	41 – 50 years	19.8
	51 – 60 years	11
	61 years and above	4.9
Gender	Male	95.6
	Female	4.4
Education	Illiterate	15.4
	Primary	31.9
	Xth	13.2
	+2 and above	39.6
Marital Status	Unmarried	25.8
	Married	74.2
Family Style	Nuclear	79.7
	Joint family	20.3
Number of Members in Your Family	Up to 2	5.5
	3 – 5	72.5
	6 – 8	19.2
	Nine and above	2.7

Source: Author’s analysis.

**Table 2.** Economic factors.

Economic factor		
		Percentage %
Average annual net income per Acres	Below Rs. 5,000	9.9
	Rs.5000 – Rs. 10000	15.4
	Rs. 10000 – Rs. 15000	52.2
	Rs. 15000 and above	22.5
Source of finance	Own fund	26.9
	Borrowed funds	20.3
	Both	52.7
Sources of borrowings	Commission agents	72.5
	Moneylenders	16.5
	Commercial banks	11

Source: Author’s analysis.

Not only that, but the research highlights how essential commission agents are to making borrowing easier because, for 72.5% of farmers, they are their primary source of funding. By serving as middlemen, these agents can buy jasmine directly from growers. These economic observations highlight how vital jasmine growing is to farmers’ livelihoods and how important it is to have various funding options made possible by commission agents to keep agricultural operations going in Huvina Hadagali.

Table 3 offers essential insights into Huvina Hadagali’s labour and employment dynamics, particularly about the demand for permanent workers in jasmine cultivation and the sufficiency of labour available. The statistics show that a sizable majority of respondents (52.2%) think there is not enough labour in the current workforce to meet the demands of jasmine production and that labour storage is an issue in this industry.

**Table 3.** Labour and employment.

Labour and employment		
		Percentage %
Opinion on existing labour	Insufficient	52.2
	Sufficient	47.8
Need permanent labour	Yes	75.8
	No	24.2
Able to reach the required labour	Yes	83
	No	17
Nature of work assigned to permanent labour	Jasmine-related works	86.8
	Other crop works	6.6
	Family works	6.6

Source: Author’s analysis.

As confirmed by 75.8% of respondents, there is an evident requirement for permanent labour because jasmine growing is a year-round endeavour. However, just 83% of respondents said they could find the necessary labour, suggesting that there may not be enough workers to meet this demand. Moreover, 84.8% of respondents indicated that most tasks given to permanent labourers are related to jasmine. This emphasises the need for labour and specialisation to meet the demands of jasmine farming. The data, taken as a whole, emphasises Huvina Hadagali’s difficulties in satisfying the demands of jasmine cultivation due to labour scarcity. It emphasises how important it is to develop practical solutions to deal with these problems and ensure that workers are prepared to handle the demands of this vital industry.

Table 4 offers a significant understanding of Jasmine’s sales and marketing dynamics in Huvina Hadagali. It clarifies the jasmine flower sales method, commission fees, the function of the blower market, and the travel time to the market. According to the research, a sizable portion of producers—61 per cent—choose to sell their jasmine flowers directly to shops via commission agents. This illustrates how standard a specific distribution route is in the neighbourhood market. Moreover, 36.3% of growers opt to sell their harvest through commission brokers to wholesalers, suggesting it is another important sales channel. Significantly, most of the jasmine grown in Huvina Hadagali is transported over 60 kilometres to the market, emphasising the value of long-distance networks in helping producers access markets.

**Table 4.** Market and sales.

Market and sales		
		Percentage %
How flowers are sold	To pre-harvest contractors	1.1
	Direct to consumer	1.6
	Direct to the retailer through a commission agent	61
	Direct to wholesaler/commission agent	36.3
Commission charges	Per kg	57.1
	Per bud	42.9
Flower market ownership	Private party	57.1
	Municipality	41.2
	Association	1.6
Market distance	1-20 km	3.3
	20-40 km	1.6
	40-60 km	7.1
	60 and above	87.9

Source: Author’s analysis.

In Huvina Hadagali, private ownership makes up most of the market ownership, as 57.1% of respondents indicated, compared to 41.2% who indicated municipal ownership. The distribution ownership underscores the involvement of various stakeholders in managing and operating the market. Therefore, the data suggests that producers rely on commission agents for sales transactions, long-distance transportation is crucial for accessing markets, and a diverse range of stakeholders owns the flower market in Huvina Hadagali.

Table 5 provides valuable insight into the post-harvest handling process of jasmine growers in e Huvina Hadagali. The focus is on value-adding transportation and the associated costs. According to the data, a significant proportion of growers - 91.2% - engage in value-addition activities, such as garland making, to enhance the market value of their jasmine. Interestingly, 7.1% of jasmine production is directed towards accessing untapped markets where commercial Hadagali Jasmine is readily available. This indicates that the growers are strategically expanding their customer base and maximising their sales opportunities. Regarding transportation, rented taxis are the primary mode of transportation at 44.5%, followed by buses at 24.7%. This reliance on rented vehicles highlights the importance of efficient transportation networks in connecting producers to markets and ensuring the timely delivery of perishable goods.

**Table 5.** Post harvest handling.

Post harvest handling		
		Percentage %
Value addition undertaken by the farmer	Garland making	91.2
	Colouring the flower	1.1
	Reaching the untouched market	7.1
The average cost incurred for transportation	a) 0-20	59.3
	b) 20-40	9.9
	c) 40-60	6.6
	d) 60-80	7.1
	e) 80 and above	17
Mode of transportation to plucked jasmine	Own vehicle	28
	Taxi cars	44.5
	Trucks	2.7
	Bus	24.7

Source: Author’s analysis.

The data also reveals the costs associated with transportation, with most growers (59.3%) incurring transportation costs ranging from up to Rs. 20 per kg. Table 5’s observations illuminate the efforts made by jasmine producers to raise the value of their crop and increase their market share.

A detailed analysis of the cultivation of jasmine by Huvina Hadagali farmers can be found in Table 6. The study looks at farmer experience level, plant life duration, degree of cultivation, and generational engagement in jasmine. According to the data, approximately 85.2% of farmers continue to conduct agriculture, passed down through family generations. This suggests that the neighborhood has a long-standing agricultural tradition. However, a discernible downturn in the growth of jasmine indicates a move away from this formerly significant farming practice. The research shows that over half of the farmers still grow jasmine on up to one acre of land, notwithstanding this drop. Some oversee more extensive regions but in smaller amounts. Furthermore, many farmers’ jasmine plants enjoy extended lifespans—49.5% 16 years. This suggests that the practice is enduring and environmentally sound. A wide range of skills is shown in the distribution of farmers’ experience levels in jasmine production; 51.6% have up to ten years of experience. The advancement and continuation of jasmine cultivation techniques are facilitated by the mix of new practitioners and their experiences. In conclusion, the research emphasises how farmers have a long-standing practice of passing down agricultural knowledge to their offspring, how the jasmine cultivation industry is evolving, and how different experience levels will shape its future.

**Table 6.** Jasmine cultivation.

Jasmine cultivation		
		Percentage %
Involvement in agriculture	Heredity	85.2
	First-generation farmer	14.8
The area under jasmine cultivation (in acres)	Up to 1	47.8
	1.1 – 2	26.9
	2.1 – 3	13.7
	3.1 and above	11.5
Average life of jasmine plant	Up to 5 years	7.7
	6 – 10 years	19.8
	11 – 15 years	23.1
	16 years and above	49.5
Experience in jasmine cultivation	Up to 10 years	51.6
	11 – 20 years	26.9
	21 – 30 years	9.3
	31 – 40 years	9.9
	41 years & above	2.2

Source: Author’s analysis.

### 4.2 Cluster analysis

The findings of a two-step cluster analysis by 364 individuals from the Vijayanagara district’s Huvina Hadagali are shown in Table 7. The study considered factors including farming practices, irrigated regions, and agricultural equipment to classify hadagali jasmine farming according to the yield produced per acre. The results show seven different clusters with sizes ranging from 11% to 19.2%, each of which represents a different percentage of the sample. Specific input variables define each cluster, such as the irrigated area, agricultural equipment, and farming techniques ( Table 10). Remarkably, the irrigated area is the most significant variable—it occurs in every cluster at 100%.

**Table 7.** Cluster distribution.

Cluster	1	2	3	4	5	6	7
Size	15.4% (56)	11% (40)	19.2% (70)	13.7% (50)	14.3% (52)	13.2% (48)	13.2% (48)
Input	Area irrigated 2 (100%)	Area irrigated 2 (100%)	Area irrigated 2 (100%)	Area irrigated 2 (100%)	Area irrigated 2 (100%)	Area irrigated 2 (100%)	Area irrigated 2 (100%)
	Type of Agri-Equipment 4 (100%)	Type of Agri-Equipment 1 (40%)	Type of Agri-Equipment 4 (100%)	Type of Agri-Equipment 4 (100%)	Type of Agri-Equipment 1 (100%)	Type of Agri-Equipment 4 (100%)	Type of Agri-Equipment 1 (100%)
	Farming method adopted. 2 (100%)	Farming method adopted. 2 (100%)	Farming method adopted. 2 (100%)	Farming method adopted. 2 (100%)	Farming method adopted. 1 (100%)	Farming method adopted. 1 (100%)	Farming method adopted. 1 (100%)

Source: Author’s analysis.

From the Table 8, a 19.2% of the sample total, the third cluster is the most significant fraction, followed by the first, fifth, fourth, seventh, and second clusters. It is worth noting that clusters two and six are relatively small and were excluded from further comparison analysis due to their limited representation in the sample ( Hennig et al., 2015). The distribution of clusters provides valuable insights into the diverse agricultural practices and characteristics observed among jasmine farmers in Huvina Hadagali, offering an understanding of the factors influencing the yield outcomes per acre ( Table 9).

**Table 8.** Farming method adopted.

Farming adopted					
		Mixed		Single	
		Frequency	Percent	Frequency	Percent
Cluster	1	0	0.0%	56	25.9%
	2	0	0.0%	40	18.5%
	3	0	0.0%	70	32.4%
	4	0	0.0%	50	23.1%
	5	52	35.1%	0	0.0%
	6	48	32.4%	0	0.0%
	7	48	32.4%	0	0.0%
	Combined	148	100.0%	216	100.0%

Source: Author’s analysis.

**Table 9.** Type of agricultural equipment used.

Type of agricultural equipment used									
		Tractor		Cutter		Weeder		Sprayer	
		Frequency	Percentage	Frequency	Percentage	Frequency	Percentage	Frequency	Percentage
Cluster	1	0	0.0%	0	0.0%	0	0.0%	56	27.2%
	2	16	13.6%	16	53.3%	8	80.0%	0	0.0%
	3	0	0.0%	0	0.0%	0	0.0%	70	34.0%
	4	0	0.0%	0	0.0%	0	0.0%	50	24.3%
	5	52	44.1%	0	0.0%	0	0.0%	0	0.0%
	6	2	1.7%	14	46.7%	2	20.0%	30	14.6%
	7	48	40.7%	0	0.0%	0	0.0%	0	0.0%
	Combined	118	100.0%	30	100.0%	10	100.0%	206	100.0%

Source: Author’s analysis.

**Table 10.** Area under irrigation.

Area under irrigation (in acres)									
		Upto 1		1.1-2		2.1-3		3.1 and above	
		Frequency	Percentage	Frequency	Percentage	Frequency	Percentage	Frequency	Percentage
Cluster	1	0	0.0%	56	53.8%	0	0.0%	0	0.0%
	2	16	17.0%	12	11.5%	2	2.3%	10	12.5%
	3	0	0.0%	0	0.0%	30	34.9%	40	50.0%
	4	50	53.2%	0	0.0%	0	0.0%	0	0.0%
	5	0	0.0%	26	25.0%	0	0.0%	26	32.5%
	6	28	29.8%	10	9.6%	6	7.0%	4	5.0%
	7	0	0.0%	0	0.0%	48	55.8%	0	0.0%
	Combined	94	100.0%	104	100.0%	86	100.0%	80	100.0%

Source: Author’s analysis.

**Figure 1.**
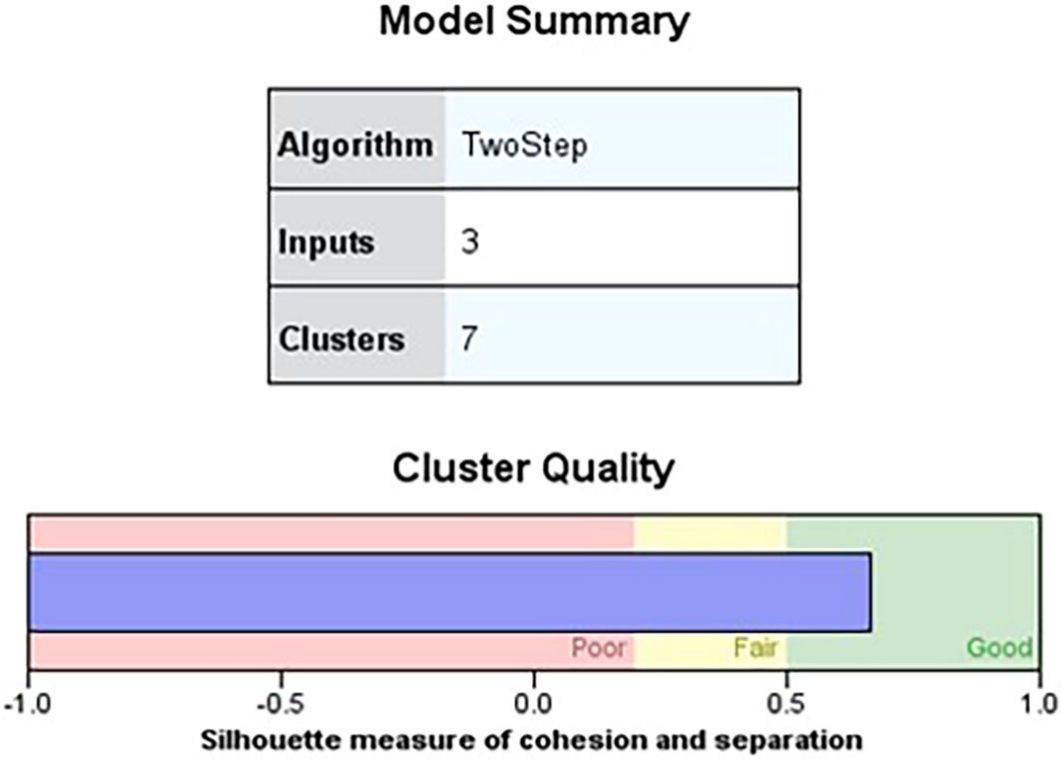
Model summary. Source: Author’s analysis.

In the 2-step cluster analysis, Table 7 displays the categorical variable and its corresponding prediction relevance, categorised into seven clusters, each with a unique pattern within the data. Meanwhile, Figure 1 showcases the quality of the cluster analysis based on three independent variables, indicating a favourable silhouette measure of cohesiveness and separation above the 0.7 threshold for good quality. Furthermore, the silhouette measure of cohesiveness and separation exceeding 0.5 suggests a favourable zone for cluster quality ( Cai et al., 2024; Hennig et al., 2015). The analysis reveals considerable differences among the independent factors, with the type of agricultural equipment used, irrigated land, and type of farming accessing individual clusters. However, respondents in all groups primarily engage in jasmine cultivation.

Table 1 outlines the order of input variables based on their significance within each cluster. In the Figure 3 and Figure 4, Clusters One, Four, and Seven significantly impact irrigated land, while Clusters Two, Three, Five, and Six deem the impact of irrigated areas insignificant. Clusters Three and Five are distinguished by their unique cultivation practices, including using specific agricultural equipment and adopting different farming methods. Conversely, Clusters Two and Six exhibit statistical significance primarily about the farming methods employed, with other factors such as irrigated area and agriculture equipment type demonstrating lesser importance.

**Figure 2.**
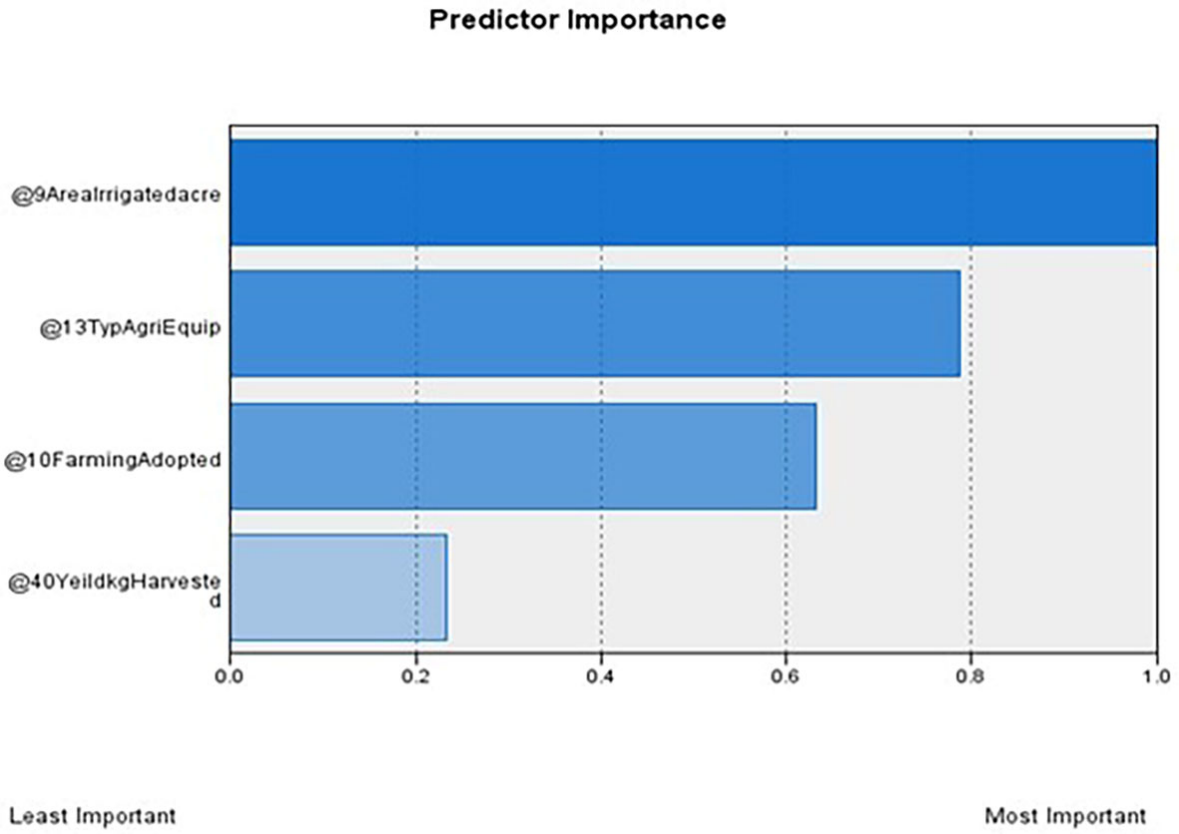
Predictor importance. Source: Author's analysis.

**Figure 3.**
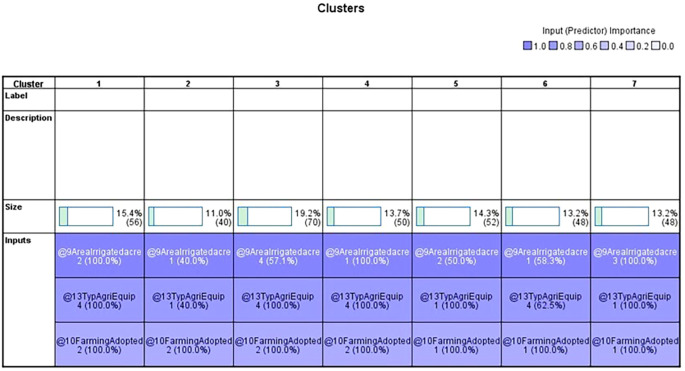
Categorical variables. Source: Author’s analysis.

**Figure 4.**
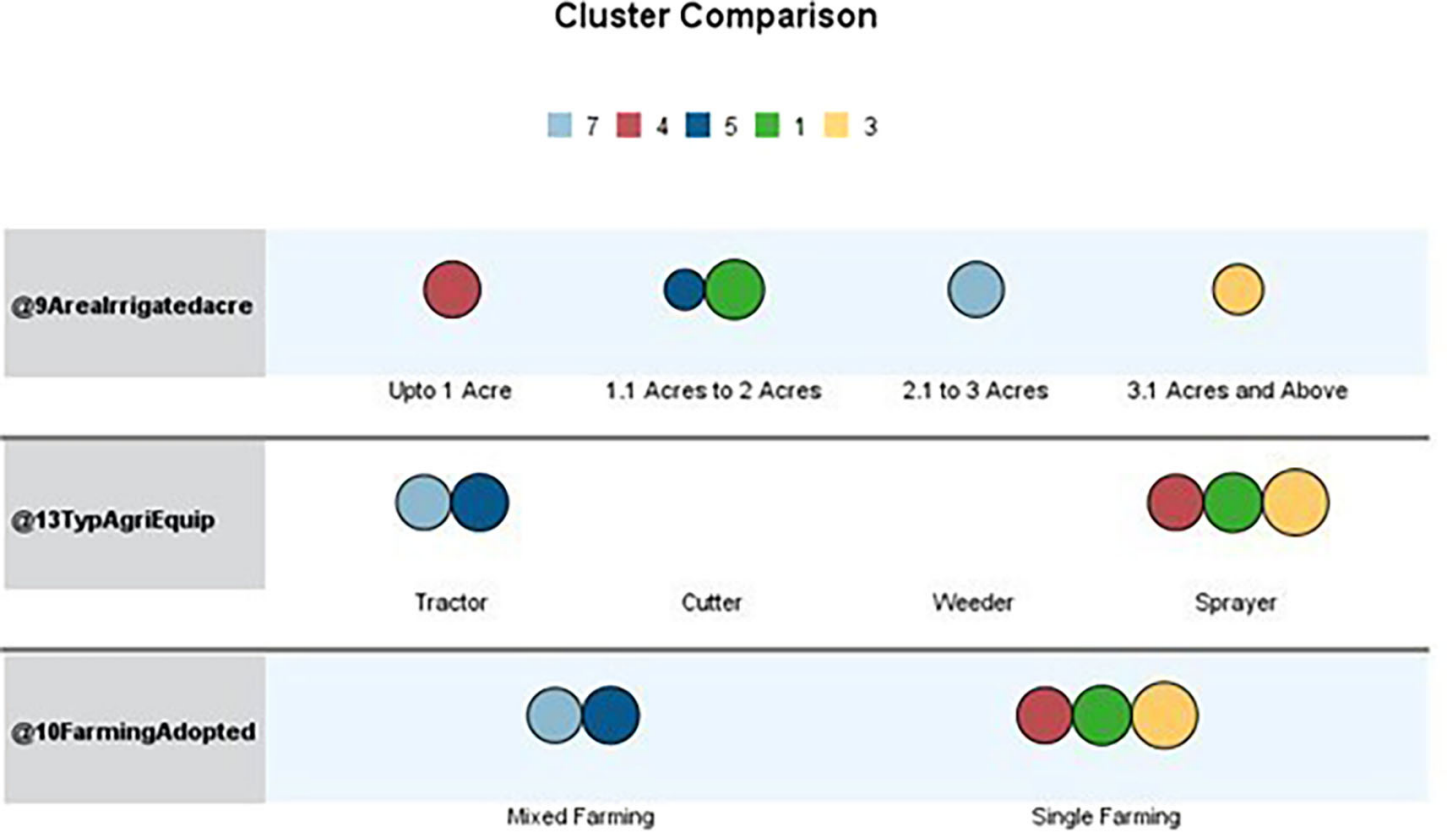
Cluster comparison. Source: Author's analysis.

Overall, the analysis offers valuable insights into the distinct pattern of jasmine cultivation practices observed across various clusters, highlighting the significance of different input variables in shaping the patterns.

The data table showcases various farming practices and agricultural equipment usage across clusters. Upon closer examination, it becomes apparent that three clusters engage in mixed cropping while the others primarily practice monoculture farming. The sample includes individuals who practice both mixed and monoculture farming and those who exclusively practice monoculture farming.

Regarding agricultural equipment, clusters two, five, six, and seven predominantly use tractors. Cluster Six stands out for its diversified use of farming equipment. In contrast, clusters one, three, and four are characterised by the farmers’ exclusive use of sprayers. The majority of farmers rely on sprayers for their agricultural activities.

Upon analysing the clusters of irrigated areas, we have discovered distinct patterns among farmers based on the size of their holdings. Farmers in the first cluster generally have between 1.1 and 2 acres of irrigated land. The second cluster includes those with holdings ranging from less than one acre to 3.1 acres or more, all under irrigation. Cluster three consists of farmers with 3.1 acres or more under irrigation. The fourth cluster comprises farmers in clusters five and six, with irrigated land ranging from 1.1 to 2 acres and 3.1 acres and above. The sixth cluster’s farmers have irrigated land ranging from less than 1 acre to 3.1 acres or more, with most of the concentration in the 1.1 to 2-acre range. Finally, the seventh cluster comprises farmers with irrigated land between 2.1 and 3 acres. It’s worth noting that most farmers tend to have between 1.1 and 2 acres of irrigated land.

After analysing the characteristics of the surveyed farmers, we have identified several distinct patterns. Cluster one (Green) comprises farmers with irrigated land holdings between 1.1 and 2 acres who mainly practice single-crop farming and rely on sprayers for crop management. This cluster has the highest number of farmers in this land size range. Cluster three (Yellow) includes farmers with irrigated land of 3.1 acres or more who prefer a single-crop farming strategy and utilise sprayers as their primary farming equipment. Cluster four (Red) consists of farmers with smaller irrigated land holdings, up to one acre, who adopt a single-crop approach and favor sprayers.

Similar agricultural practices are demonstrated by Clusters Five (Royal Blue) and Seven (Sky Blue), which use only mixed farming and mostly tractors as their primary equipment. Nonetheless, Cluster Seven and Cluster Five have different land holdings; Cluster Seven owns 2.1 to 3 acres of irrigated land, whereas Cluster Five has 1.1 to 2 acres. Overall, it is clear that the clusters that have been discovered differ in terms of farming practices and agricultural equipment. Notably, none of the farming practices in the clusters involve mowers or weeders. The variations in land size, crop selection, and equipment preferences among the clusters underscore the heterogeneity in the agricultural community under study.

## 5. Discussions

In the constantly changing field of agriculture, particularly in Huvina Hadagali, the combination of tradition and innovation offers a unique potential and challenge. The local government must uphold this delicate balance and be guardians of the land and its agricultural heritage. While welcoming the winds of change blowing across the industry, preserving the fabric of farming methods is imperative.

Initiatives to educate farmers about the advantages of contemporary farming methods are at the center of this endeavor. Farmers can obtain valuable insights into the potential benefits of implementing sophisticated technologies and optimising irrigation methods through focused education campaigns and outreach programs. Local governments can empower farmers to make well-informed decisions regarding their livelihood by eliminating impediments to modernisation, such as high investment costs and operational complexity.

Another important factor in easing this shift to sustainability is financial support. By facilitating access to reasonably priced finance and offering incentives for adopting contemporary machinery and technologies, local governments may lessen the financial strain on farmers and encourage investment in innovation. Furthermore, farmers can pool their resources and benefit from economies of scale by using cooperative farming methods and collective negotiating power, increasing their market competitiveness. But it’s important to remember the inherent worth of tradition in the middle of the drive for advancement. Deeply ingrained in Huvina Hadagali’s cultural legacy, the production of jasmine is a moving reminder of the unbreakable bond between the land and its people. Local government bodies may ensure that farmers maintain the spirit of tradition by encouraging a sense of pride and continuity among them through the preservation and celebration of these traditional traditions.

## 6. Conclusion

It’s utmost importance on our part to understand and support the farmers who serve society in various forms. The region, of our study is recognised by the produce of Jasmine, and hence, farmers feel proud about their livelihood. However, time and preferences are changing, leading to lots of socio-economic dimensions related to jasmine cultivation is also experiencing turbulence. Inexperienced farmers, low yield, lack of mechanisation moreover uncertainty in their income generation are typically the features of farming community in Hadagali.

Rebuilding of lost reputation and injecting new blood into this holy work of floriculture needs lots of government intervention. Even though there exist policies, farmers are unable to reap the benefits due to political willpower of representatives and thus government unable to reach the needy. Academician’s work needs to be geared up with field-based study, giving bit of moral strength to the community at large

### Ethics and consent statement

This research has been conducted in strict accordance with existing ethical criteria due to its involvement with human interactions. It has obtained the necessary ethical clearance (Institutional Ethics Committee (IEC) at Kasturba Medical College and Kasturba Hospital, Manipal, Karnataka, India- issued on 14 ^th^ December 2021, indicating that “project comes seems to come under pure economic, social and managerial research. If there are no health-related aspects involved, then you may proceed without IEC clearance” from the Institutional Ethics Committee (IEC) at Kasturba Medical College and Kasturba Hospital, Manipal, Karnataka, India. In addition, during the implementation of this non-experimental research, the researcher collected written informed consent from all participants, assuring them that their personal information would be handled with the highest level of confidentiality.

## Data Availability

The Data sheet is available in the DRYAD data repository. Availability of the data sheet details are given below. Repository Name: DRYAD - https://doi.org/10.5061/dryad.tb2rbp09d ( Joisa *et al*., 2024) The project contains the following underline data:
• Data File 1. Excel (364 coded respondents data sheet) Repository Name: DRYAD - https://doi.org/10.5061/dryad.tb2rbp09d ( Joisa *et al*., 2024) The project contains the following extended data:
• Questionnaire included in the readme file. Data are available under the terms of the Creative Commons Zero “No rights reserved” data waiver (CC0 1.0 Public domain dedication).

## Software availability statement

IBM (SPSS) Statistics version 27 is a subscribed software for Manipal Academy for Higher Education.
