# National Sentiments and Regional Flavour- A Socio-economic Study of Huvina Hadagali Jasmine

**DOI:** 10.12688/f1000research.153101.3

**Published:** 2025-12-22

**Authors:** Jyeshtaraja Joisa, Harisha G Joshi, Kavitha T C, Javed Bhasha

**Affiliations:** 1 Department of Commerce, Manipal Academy of Higher Education, Manipal, Karnataka, 576104, India; 2 Department of Commerce, GANGAVATHI BHAGYAMMA RURAL DEGREE COLLEGE, HUVINHADAGALI, Huvina Hadagali, Karnataka, 583219, India

**Keywords:** Hadagali Jasmine, Socio-economic study, 2-Step cluster analysis, Floriculture, Jasmine flower, floriculture market.

## Abstract

**Background:**

This socio-economic analysis studies the influence of jasmine production on the economic well-being of farmers in Huvina Hadagali, a region known for its high-quality jasmine flowers. The Vijaya Nagara district’s Havina Hadagali area is well known throughout the country for its jasmine flower farming. In addition to being referred to as Mallige Nadu, this location is also known as Malligeya Tavaru. The cultivation of the jasmine flower is protected by the Geographical Indication (GI) Tag, and this flower has been popular in this region for a substantial amount of time.

**Methods:**

Data was collected from a sample of 364 jasmine growers using a structured questionnaire in Huvina Hadagali, Vijayanagar district. The data focused on different socio-economic factors such as income levels, employment, market access, and agricultural techniques. The study is analysed using IBM SPSS through frequency analysis and 2-step clustering.

**Result:**

The results demonstrate that the cultivation of jasmine makes a substantial contribution to the local economy, serving as a main or additional source of income for numerous households. Jasmine farming often contributes 40% of the whole household income, and during peak seasons, it provides significant economic advantages. Nevertheless, the highlighted obstacles were volatile market pricing, pest infestations, and limited access to contemporary farming practices. Due to their decades of experience, it is felt by the farmers. Hadagali Jasmine comes under the GI Tag, hence they are authorised farmers. The study emphasises the crucial significance of cooperative societies and local marketplaces in stabilising income and offering essential resources and training to farmers.

**Conclusion:**

The research highlights the necessity of governmental interventions focused on developing market infrastructure, offering financial assistance, and improving access to agricultural innovations to maintain and augment the economic advantages of jasmine cultivation in Huvina Hadagali which also lead to an overall development of floriculture in India.

## 1. Introduction

India has a long-standing association with flowers, as shown by its use in ancient texts, traditions, and paintings ( Bhattacharya, 2014). Rigveda, an old sacred piece of literature, acknowledges the significance of flowers in ceremonies and everyday life. Flowers have several applications. Flowers’ spiritual and aesthetic importance is emphasized in Rigveda ( Balkrishna et al., 2019). Flowers play an essential role in both ceremonies and daily activities. The development of floriculture in India has been significantly influenced by several dynasties and civilizations throughout its history. These dynasties and cultures have improved floriculture by introducing new species and innovative cultivation methods ( P.Renganathan & Dr. A. Gopalakrishnan, 2019). The large terrain of India, which is rich in cultural legacy and floral practices, yields a diverse range of flowers planted across the country. Patel et al. (2022) highlighted that the captivating fragrance and cultural significance of jasmine set it apart from other popular flowers. In Indian tradition, jasmine is revered as a sign of purity, grace, and welcome. As a subfield of horticulture, floriculture is a specialized field that focuses on cultivating attractive blooming plants ( Latha R & Dr. R. Pichumani, 2018). These plants are grown for commercial use, such as selling or as raw materials for the cosmetics industry. There has been a constant increase in demand for floricultural products in both the home and international markets ( Ganga et al., 2019). Cut flowers, which have great potential for worldwide trade, have resulted from India’s significant progress in flower agriculture ( Carton & Parigot, 2022).

Farming flowers and decorative plants in India addresses various activities, including cultivation, processing, retailing, and exporting these products. India is an ideal floriculture destination throughout its regions because of its varied agro-climatic zones, excellent environment, and abundant competent labor ( Swain & Maurya, 2021). The gorgeous white blossoms and entrancing smells that become more intense throughout the nighttime hours of this floral fragment, which belongs to the genus Jasminum, are the reasons for its great worth. It is believed that jasmine has been cultivated in India for a very long time, reaching back to ancient times. Allusions from classical works, poetry, and folklore Balkrishna et al. (2019) make this point abundantly clear. India is one of the world’s largest jasmine-growing countries; major cultivation areas are found in parts of Maharashtra, Uttar Pradesh, West Bengal, and the Southern States of Tamil Nadu, Karnataka, Andra Pradesh, and Kerala ( Yoganandan, 2020). It is possible to cultivate jasmine in an excellent environment thanks to favorable climatic conditions, which include high temperatures, moderate precipitation, and soil that drains well. According to Palanisingh et al. (2022), Karnataka, situated in southern India, is delighted because it cultivates various colours and flowers. This flourishing sector is deeply rooted in the state’s cultural heritage and can be attributed to the ideal agricultural and climatic circumstances. In this detailed inquiry, the various landscapes of floriculture in the state of Karnataka are investigated. Particular attention is paid to the growing methods utilised and Jasmine’s economic worth and cultural significance. Karnataka has emerged as a significant centre for floriculture in India, with a flourishing industry that includes the whole process, from cultivating to selling cut flowers and decorative plants ( Yakandawala, 2016).

Commercial cultivation of jasmine in eastern India serves both domestic and international markets, with a significant portion of the production consumed locally for religious and cultural purposes ( Landran & Gupta, 2017). Jasmine is a flower belonging to the jasmine flower group. According to Cnaan et al. (2014), the jasmine flower market is dynamic and influenced by several factors such as festivals, weddings, seasonal demand, and religious occasions. While traditional channels such as neighborhood flower markets, houses of worship, and florists remain essential sales channels, e-commerce has opened new opportunities for speaking with customers directly ( D’souza & Joshi, 2020). India’s agricultural economy has benefited significantly from the proliferation of jasmine, which has had a substantial economic impact. This is profitable for small-scale or economically disadvantaged farmers, particularly those who rely on jasmine production as their primary source of income. The Vijaya Nagara district’s Havina Hadagali, comprising of 23 villages are well-known throughout the country for jasmine flower farming. In addition to being referred to as Mallige Nadu, this location is known as Malligeya Tavaru. Approximately 1000 families depend upon jasmine cultivation and it is the major source of their income. The Hadagali jasmine flower is protected by Geographical Indication (GI) tag (2008) , and this flower has been popular in this region for a substantial amount of time. To determine the socio-economic standing of jasmine producers, farmers are engaged in the cultivation of G.I.-tagged jasmine ( Nagaveni, 2018). It is essential to investigate the history of Hadagali Jasmine and understand various value additions to the existing farm practices in order to enhance the socio-economic profile of the farmers.

## 2. Literature review

Floriculture is a subfield of horticulture that focuses on the cultivation, marketing, and aesthetic arrangement of flowers and decorative plants for commercial purposes ( Krishnaveni & Pethalakshmi, 2017). This includes the cultivation of annuals, biennials, and perennials ( Anumala & Kumar, 2021). Plant-cut foliage, seed bulbs, tubers, rooted cuttings, dried flowers, or leaves primarily comprise blooms removed from the plant ( Beneragama & Peiris, 2016). The transition from traditional crops to high-value-added crops, such as horticulture and floriculture, is gradually occurring in economically developing South Asian countries ( Saripalle, 2016). India’s youth benefit from increased revenue, profits, and employment prospects thanks to the floriculture industry, which also supports the participation of women to a substantial degree and boosts exports ( Krishnaveni & Pethalakshmi, 2017). Notable breakthroughs have been made in India’s flower cultivation, particularly in creating blooms with extensive export potential. This industry creates work opportunities and generates higher incomes for women ( Anumala & Kumar, 2021). In terms of floriculture, India has a long history, resulting in a significant number of advantages. Agricultural products and trade are essential to India’s economy. It has been decades since India’s small and marginal farmers could lessen their pricing risk owing to improved agricultural marketing ( Agrawal et al., 2022; Markelova et al., 2009). Flowers are commodities with concise shelf lives. Thus, marketing is a significant challenge. Rural areas are responsible for producing flowers, whereas urban areas are the principal consumers of these flowers. There is a need for flowers in rural areas only for special events such as weddings and festivals. Therefore, growers must locate markets in metropolitan areas. Only a few types of flowers, including rose gladiolus, chrysanthemums, jasmine, and orchids, have been produced and exported from India ( Lepcha et al., 2020).

One of the most essential ornamental and flavorful plants, jasmine, has been traditionally cultivated in several countries. The production of essential oils as “concrete” and “absolute” is another application. These oils are used in cosmetic and perfume industries ( Ray et al., 2014). Fully open flowers contain the highest possible aroma and must be harvested to extract concrete. Since ancient times, small farmers have traditionally sold their crops to intermediaries at the farm gate, frequently at low prices ( Gills et al., 2017). As a result of their heavy reliance on wholesale brokers and traders for market knowledge and credit facilities, most small and medium-sized farmers in these nations are bound to informal contractual arrangements. A manual classification system was used to determine the flower quality ( Krishnaveni & Pethalakshmi, 2017). The freshness of the flowers is diminished during this process, which takes a significant amount of time. As a result, judging the quality of products has become extremely difficult. Considering that these tasks are performed manually and require significant labor, it is essential to automate the determination of flower quality ( Thakur et al., 2023).

Agricultural producers worldwide, including those in India, face significant challenges from market-related constraints, such as the marketing and supply chain of agricultural produce ( Yapa, 2022). Over the past few years, one of the most critical concerns raised in India has been the effectiveness of the marketing of agricultural products. Additionally, a smaller proportion of consumer rupees are reaching growers, which is regarded as the fundamental reason for high and volatile consumer prices. Inadequate marketing infrastructure and the poor effectiveness of marketing channels are also believed to be related to this issue ( Sathish & Rajamohan, 2019). During COVID-19, there was a shortage of laborers because of restrictions placed on migrant workers. Although the consumer purchase rate is high, the number of agricultural commodities produced by farmers is low ( Sandeep Kumar et al., 2020). In the context of a changing climate and diminishing land resources, water scarcity, pests, and illnesses, an ever-increasing population, low productivity under open conditions, and changes in consumer choice are the causes that drive more people to switch to protected cultivation ( Pachiyappan et al., 2022). Land tenure plays a significant role in the adoption and implementation of erosion control measures ( Sohal et al., 2023). This is especially true for technologies that require extensive planning and commitment over extended periods. Most farmers who grow flowers sell their produce to commission agents in the flower mandi, without adding value. The economic well-being of flower producers needs to have post-harvest management and value-addition programs in place within the floriculture industry. The introduction of novel marketing arrangements can potentially alter market relations in a manner that serves smallholders’ interests ( Gills et al., 2017).

Producer organizations are in an advantageous position to capitalize on these novel methods. In addition to displaying a great interest in agricultural technology, young people who are unemployed and educated, but have no interest in traditional agriculture, also exhibit a strong interest in developing agricultural technologies ( Thakur et al., 2023). Additionally, it has been noted that the costs associated with the protected cultivation of floral crops are higher than the yield realized from the flowers ( Pachiyappan et al., 2022). It is important to note that specific technology categories, such as enhanced varieties and chemical input, have a higher possibility of being adopted on larger farms, raising questions about the scalability of these technologies. Nevertheless, it is quite different from tradition in assessing the degree to which farmers encounter credit constraints, instead of merely determining their credit accessibility. This is even though the relevance of agricultural finance is obvious: the implementation of a higher order. Compared to deploying a single technology, integrating multiple technologies results in more significant dividends for farmers regarding form, yield, and income ( Nsabimana & Adom, 2024). Various integrated technologies were investigated, and the findings revealed that technological mining, involving crop and soil improvements, had the most substantial influence. Crop intensification projects aim to achieve sustainable improvement in productivity by acquiring improvised seeds, fertilizers, and pesticides to increase crop production potential. With population expansion, climate change, rising demand for high-quality produce, dwindling band holdings, and significant pressure on resources, there is a duty to employ modern crop production methods for protected cultivation ( Pachiyappan et al., 2022). This obligation is one of the reasons why protected cultivation is important. Protected agriculture is a high-tech method that allows crop production in an environment that is controlled and protected from bad climatic conditions. Protected cultivation is more sustainable than open cultivation methods, as the impact of climate is significantly reduced because the environment is controlled. Additionally, the inputs, which include fertilizers, pesticides, and water, are utilized more effectively than in open cultivation methods. Furthermore, the increased productivity and improved quality of the produce ensure a higher return on investment. When it comes to protected cultivation, the implementation of contemporary technologies, such as artificial intelligence, robotics for plucking, and other similar technologies, as well as the utilization of the Internet of Things (IoT) or sensor-based irrigation scheduling, would contribute to an increase in both efficiency and income for farmers in the region. To support protected gardening more, the Goods and Services Tax (GST) on playhouses and warehouses in India must be lowered ( Anumala & Kumar, 2021). This should also be done for precision farming, value addition, and the creation of an integrated flower cold chain. The government of India has determined that floriculture is a sunrise industry and has been given the status of being entirely export-focused. As a result of the consistent rise in demand for flowers, floriculture has emerged as one of the most important commercial business sectors in the agricultural sector. Over the previous few decades, the topics of ecological tourism, emerging mode, and rural regeneration have been the focus of studies that have been conducted for a considerable amount of time. Agricultural practices that are based on ecological principles and emphasize environmental sustainability are referred to as ecological agriculture, also called eco-agricultural practices ( Fan et al., 2024).

The proliferation of governance mechanisms, such as agricultural contracts, farmers’ groups, estate farming, and farm non-governmental organizations, further drives the change by improving vertical integrational horizontal coordination and, as a result, making it easier for small farmers to access high-value markets ( Jagadeesh, 2020). High market value participation suits family income, productivity, economic growth, and development in rural areas. For this reason, developmental policymakers need to understand the patterns of high market value participation ( Ola & Menapace, 2020). In addition to filling in coordination gaps, market access can be improved through collaboration, which can help correct some imperfections in the market, such as excessive transaction costs and the absence of credit markets ( Ola & Menapace, 2020). Furthermore, when farmers combine their financial and labor resources, they are better able to gather the necessary information, meet quality standards, and operate on a large scale. This allows them to sell their products to new local or foreign markets, which would otherwise be out of reach for smaller producers ( Shah et al., 2022).

## 3. Study design

### 3.1 Methods

The primary aim of this study was to investigate various aspects of jasmine cultivation, focusing on cultivators in the Huvina Hadagali region. The study employed a questionnaire-based approach to gather data from jasmine cultivators, with subsequent analysis utilizing frequency and percentage analysis and two-step cluster analysis using IBM SPSS. A cross-sectional research design was adopted to collect data from jasmine cultivators in the Huvina Hadagali region. This study employed a purposive sampling technique to select participants actively involved in jasmine cultivation in the Huvina Hadagali region. Purposive sampling allows researchers to choose participants who are most relevant to the research objectives, ensuring that the sample closely aligns with the study’s aims ( Campbell et al., 2020; Memon et al., 2025). This targeted approach can enhance the rigor and trustworthiness of data and results ( Memon et al., 2025). It is also used when the researcher has no access to the population or when the population is indefinite. One advantage of purposive sampling is that it facilitates the establishment of trust and rapport with participants, which is essential for obtaining honest and meaningful data ( Uddin & Paul, 2023). The sample size for this study was determined to be 364 jasmine cultivators from Huvina Hadagali. A structured questionnaire was developed to collect data from the participants. The questionnaire consisted of closed-ended questions covering various aspects related to jasmine cultivation, including cultivation practices, challenges faced, yield, market access, and socio-demographic characteristics of the cultivators. A pilot study was conducted before data collection to ensure the relevance and reliability of the research instruments. This pilot study involved a subset of the target population, allowing for the identification of ambiguities, inconsistencies, and potential biases in the questionnaires. Additionally, the questionnaire was validated by experts to establish its content validity. A panel of experts in floriculture was consulted to review the questionnaires. The combined use of a pilot study and expert validation strengthened the instrument’s validity and ensured that it was both comprehensible to respondents and capable of producing meaningful and credible data aligned with the objectives of the study. Reliability and construct validity were omitted as the focus of the study was on exploitative segmentation analysis rather than on a theoretical testing investigation. After collecting the questionnaire responses, the data were subjected to frequency and percentage analyses to examine the distribution of responses across different variables. This analysis provides insights into the prevalence of various practices, challenges, and characteristics among jasmine cultivators in Huvina Hadagali. Additionally, a two-step cluster analysis was conducted using IBM SPSS version 27 (Statistical Package for the Social Sciences) to identify distinct groups or clusters within the sample population, based on their responses to the questionnaire. In this study, two-step cluster analysis was employed to identify natural groupings within the dataset based on floriculture variables. The cluster selection was guided by a combination of statistical and interpretability criteria. The two-step clustering algorithm utilizes the Bayesian information criterion (BIC) to automatically determine the optimal number of clusters. Clusters were automatically selected using the software. The average silhouette coefficient was used to assess the quality of the cluster selection. A silhouette value close to +1 indicates that the data points are well matched to their own clusters and poorly matched to neighboring clusters. A silhouette measure of ≥0.5 was considered an indicator of good clustering quality. SPSS provides an average silhouette coefficient ranging from -1 to +1. A value closer to +1 indicates well-separated and cohesive clusters. A silhouette value of ≥0.5 was considered acceptable for high-quality clustering. This analytical technique helps to uncover patterns or relationships among the variables and allows for a deeper understanding of the heterogeneity within the population of jasmine cultivators.

## 4. Findings and Discussion

### 4.1 Demographic analysis

Some exciting trends can be seen in the demographic analysis of farmers in Huvina Hadagali. It demonstrates how young and active the farming community is, with a sizable percentage (36.8%) of its members under 30 years old. In addition, among the farming community’s decently educated members, a sizeable percentage (39.6%) had completed higher education beyond the twelfth grade. These insights shed light on the changing dynamics within the Havina Hadagali agricultural community, emphasizing how family structure, age, gender, and education affect group makeup and operations. The increasing number of nuclear families and the three- to five-member majority of agricultural families are signs of the community’s adaptation and resilience.

Table 2 thoroughly examines Huvina Hadagali farmers’ economic conditions, concentrating on their sources of revenue and ways of financing their farming operations. A sizable portion of farmers (52.2%) used the production of jasmine as their primary source of yearly net income per acre, with the majority making between Rs. 10000 and Rs. 15000. Interestingly, most farmers in Havina Hadagali (52.7%) used borrowed and personal funds to finance their agricultural operations. This emphasizes how the farming sector needs to diversify financially to maintain viability.

**Table 1.** Demographic factors.

Demographics factor		
		Percentage %
Age	Up to 30 years	36.8
	31 – 40 years	27.5
	41 – 50 years	19.8
	51 – 60 years	11
	61 years and above	4.9
Gender	Male	95.6
	Female	4.4
Education	Illiterate	15.4
	Primary	31.9
	Xth	13.2
	+2 and above	39.6
Marital Status	Unmarried	25.8
	Married	74.2
Family Style	Nuclear	79.7
	Joint family	20.3
Number of Members in Your Family	Up to 2	5.5
	3 – 5	72.5
	6 – 8	19.2
	Nine and above	2.7

Source: Author’s analysis.

**Table 2.** Economic factors.

Economic factor		
		Percentage %
Average annual net income per Acres	Below Rs. 5000	9.9
	Rs. 5000 – Rs. 10000	15.4
	Rs. 10000 – Rs. 15000	52.2
	Rs. 15000 and above	22.5
Source of finance	Own fund	26.9
	Borrowed funds	20.3
	Both	52.7
Sources of borrowings	Commission agents	72.5
	Moneylenders	16.5
	Commercial banks	11

Source: Author’s analysis.

In addition, this research highlights how essential commission agents make borrowing easier because, for 72.5% of farmers, they are their primary source of funding. By serving as middlemen, these agents can buy jasmine directly from the growers. These economic observations highlight how vital jasmine growth is to farmers’ livelihoods and how important it is to have various funding options made possible by commission agents to maintain agricultural operations in Huvina Hadagali.

Table 3 offers essential insights into Huvina Hadagali’s labor and employment dynamics, particularly about the demand for permanent workers in jasmine cultivation and the sufficiency of labor available. The statistics show that a sizable majority of respondents (52.2%) think there is not enough labor in the current workforce to meet the demands of jasmine production and that labor storage is an issue in this industry.

**Table 3.** Labor and employment.

Labor and employment		
		Percentage %
Opinion on existing labor	Insufficient	52.2
	Sufficient	47.8
Need permanent labor	Yes	75.8
	No	24.2
Able to reach the required labor	Yes	83
	No	17
Nature of work assigned to permanent labor	Jasmine-related works	86.8
	Other crop works	6.6
	Family works	6.6

Source: Author’s analysis.

As confirmed by 75.8% of respondents, there is an evident requirement for permanent labor because jasmine growth is a year-round endeavor. However, only 83% of respondents said they could find the necessary labor, suggesting that there may not be enough workers to meet this demand. Moreover, 84.8% of the respondents indicated that most tasks given to permanent laborers were related to jasmine. This emphasizes the need for labor and specialization to meet the demands of jasmine farming. The data, taken as a whole, emphasize Huvina Hadagali’s difficulties in satisfying the demands of jasmine cultivation due to labor scarcity. This emphasizes the importance of developing practical solutions to deal with these problems and ensuring that workers are prepared to handle the demands of this vital industry.

Table 4 provides a significant understanding of Jasmine’s sales and marketing dynamics in Huvina Hadagali. It clarifies the jasmine flower sales method, commission fees, function of the blower market, and travel time to the market. According to research, a sizable portion of producers—61 percent—choose to sell their jasmine flowers directly to shops via commission agents. This illustrates the standard of a specific distribution route in the neighborhood market. Moreover, 36.3% of growers opt to sell their harvest to wholesalers through commission brokers, suggesting that it is another important sales channel. Significantly, most jasmines grown in Huvina Hadagali are transported over 60 km to the market, emphasizing the value of long-distance networks in helping producers access the market.

**Table 4.** Market and sales.

Market and sales		
		Percentage %
How flowers are sold	To pre-harvest contractors	1.1
	Direct to consumer	1.6
	Direct to the retailer through a commission agent	61
	Direct to wholesaler/commission agent	36.3
Commission charges	Per kg	57.1
	Per bud	42.9
Flower market ownership	Private party	57.1
	Municipality	41.2
	Association	1.6
Market distance	1-20 km	3.3
	20-40 km	1.6
	40-60 km	7.1
	60 and above	87.9

Source: Author’s analysis.

In Huvina Hadagali, private ownership makes up most of the market ownership, as indicated by 57.1% of respondents, compared to 41.2% who indicated municipal ownership. Distribution ownership underscores the involvement of various stakeholders in market management and operation. Therefore, the data suggest that producers rely on commission agents for sales transactions, long-distance transportation is crucial for accessing markets, and a diverse range of stakeholders own the flower market in Huvina Hadagali.

Table 5 provides valuable insights into the post-harvest handling process of jasmine growers in Huvina Hadagali. The focus is on value-adding transportation and the associated costs. According to the data, a significant proportion of growers (91.2%) engage in value-addition activities such as garland making to enhance the market value of their jasmine. Interestingly, 7.1% of jasmine production is directed towards accessing untapped markets, where commercial Hadagali Jasmine is readily available. This indicates that growers strategically expand their customer base and maximize their sales opportunities. Regarding transportation, rented taxis were the primary mode of transportation at 44.5%, followed by buses at 24.7%. This reliance on rented vehicles highlights the importance of efficient transportation networks for connecting producers to markets and ensuring the timely delivery of perishable goods.

**Table 5.** Post harvest handling.

Post harvest handling		
		Percentage %
Value addition undertaken by the farmer	Garland making	91.2
	Colouring the flower	1.1
	Reaching the untouched market	7.1
The average cost incurred for transportation	a) 0-20	59.3
	b) 20-40	9.9
	c) 40-60	6.6
	d) 60-80	7.1
	e) 80 and above	17
Mode of transportation to plucked jasmine	Own vehicle	28
	Taxi cars	44.5
	Trucks	2.7
	Bus	24.7

Source: Author’s analysis.

The data also reveal the costs associated with transportation, with most growers (59.3%) incurring transportation costs ranging from up to Rs. 20 per kg. Table 5 illuminate the efforts made by jasmine producers to raise the value of their crops and increase their market share.

A detailed analysis of the cultivation of jasmine by Huvina Hadagali farmers is presented in Table 6. This study examined farmer experience level, plant life duration, degree of cultivation, and generational engagement in jasmine. According to the data, approximately 85.2% of farmers continue to conduct agriculture and pass it down through family generations. This suggests that the neighborhood has a long-standing agricultural tradition. However, a discernible downturn in jasmine growth indicates a move away from this formerly significant farming practice. Research shows that over half of the farmers still grow jasmine on up to one acre of land, notwithstanding this drop. Some oversee more extensive regions but in smaller amounts. Furthermore, many farmers’ jasmine plants enjoy extended lifespans (49.5%, 16 years). This suggests that the practice is enduring and environmentally friendly. A wide range of skills is shown in the distribution of farmers’ experience levels in jasmine production, with 51.6% having up to ten years of experience. The advancement and continuation of jasmine cultivation techniques is facilitated by a mix of new practitioners and their experiences. In conclusion, this study emphasizes how farmers have a long-standing practice of passing down agricultural knowledge to their offspring, how the jasmine cultivation industry is evolving, and how different levels of experience will shape their future.

**Table 6.** Jasmine cultivation.

Jasmine cultivation		
		Percentage %
Involvement in agriculture	Heredity	85.2
	First-generation farmer	14.8
The area under jasmine cultivation (in acres)	Up to 1	47.8
	1.1 – 2	26.9
	2.1 – 3	13.7
	3.1 and above	11.5
Average life of jasmine plant	Up to 5 years	7.7
	6 – 10 years	19.8
	11 – 15 years	23.1
	16 years and above	49.5
Experience in jasmine cultivation	Up to 10 years	51.6
	11 – 20 years	26.9
	21 – 30 years	9.3
	31 – 40 years	9.9
	41 years & above	2.2

Source: Author’s analysis.

### 4.2 Cluster analysis

Two-step cluster analysis segmented the jasmine cultivators into seven distinct clusters, each demonstrating unique socioeconomic and operational characteristics. These findings have significant implications for tailoring agricultural support, improving market access, and designing inclusive strategies for rural development. The findings of the two-step cluster analysis of 364 individuals from the Vijayanagara district’s Huvina Hadagali are shown in Table 7. The study considered factors including farming practices, irrigated regions, and agricultural equipment to classify Hadagali Jasmine farming according to the yield produced per acre. The results showed seven different clusters with sizes ranging from 11% to 19.2%, each of which represents a different percentage of the sample. Specific input variables defined each cluster, such as irrigated area, agricultural equipment, and farming techniques ( Table 10). Remarkably, irrigated area was the most significant variable, occurring in every cluster at 100%.

**Table 7.** Cluster distribution.

Cluster	1	2	3	4	5	6	7
Size	15.4% (56)	11% (40)	19.2% (70)	13.7% (50)	14.3% (52)	13.2% (48)	13.2% (48)
Input	Area irrigated 2 (100%)	Area irrigated 2 (100%)	Area irrigated 2 (100%)	Area irrigated 2 (100%)	Area irrigated 2 (100%)	Area irrigated 2 (100%)	Area irrigated 2 (100%)
	Type of Agri-Equipment 4 (100%)	Type of Agri-Equipment 1 (40%)	Type of Agri-Equipment 4 (100%)	Type of Agri-Equipment 4 (100%)	Type of Agri-Equipment 1 (100%)	Type of Agri-Equipment 4 (100%)	Type of Agri-Equipment 1 (100%)
	Farming method adopted. 2 (100%)	Farming method adopted. 2 (100%)	Farming method adopted. 2 (100%)	Farming method adopted. 2 (100%)	Farming method adopted. 1 (100%)	Farming method adopted. 1 (100%)	Farming method adopted. 1 (100%)

Source: Author’s analysis.

From Table 8, 19.2% of the total sample, the third cluster is the most significant fraction, followed by the first, fifth, fourth, seventh, and second clusters. It is worth noting that clusters two and six are relatively small and were excluded from further comparative analysis because of their limited representation in the sample ( Hennig et al., 2015). Clusters 1 and 3 of the entire group practiced single-crop farming, indicating limited diversification and potential vulnerability to markets or climatic shocks. The distribution of clusters provides valuable insights into the diverse agricultural practices and characteristics observed among jasmine farmers in Huvina Hadagali, offering an understanding of the factors influencing yield outcomes per acre ( Table 9).

**Table 8.** Farming method adopted.

Farming adopted					
		Mixed		Single	
		Frequency	Percent	Frequency	Percent
Cluster	1	0	0.0%	56	25.9%
	2	0	0.0%	40	18.5%
	3	0	0.0%	70	32.4%
	4	0	0.0%	50	23.1%
	5	52	35.1%	0	0.0%
	6	48	32.4%	0	0.0%
	7	48	32.4%	0	0.0%
	Combined	148	100.0%	216	100.0%

Source: Author’s analysis.

**Table 9.** Type of agricultural equipment used.

Type of agricultural equipment used									
		Tractor		Cutter		Weeder		Sprayer	
		Frequency	Percentage	Frequency	Percentage	Frequency	Percentage	Frequency	Percentage
Cluster	1	0	0.0%	0	0.0%	0	0.0%	56	27.2%
	2	16	13.6%	16	53.3%	8	80.0%	0	0.0%
	3	0	0.0%	0	0.0%	0	0.0%	70	34.0%
	4	0	0.0%	0	0.0%	0	0.0%	50	24.3%
	5	52	44.1%	0	0.0%	0	0.0%	0	0.0%
	6	2	1.7%	14	46.7%	2	20.0%	30	14.6%
	7	48	40.7%	0	0.0%	0	0.0%	0	0.0%
	Combined	118	100.0%	30	100.0%	10	100.0%	206	100.0%

Source: Author’s analysis.

**Table 10.** Area under irrigation.

Area under irrigation (in acres)									
		Upto 1		1.1-2		2.1-3		3.1 and above	
		Frequency	Percentage	Frequency	Percentage	Frequency	Percentage	Frequency	Percentage
Cluster	1	0	0.0%	56	53.8%	0	0.0%	0	0.0%
	2	16	17.0%	12	11.5%	2	2.3%	10	12.5%
	3	0	0.0%	0	0.0%	30	34.9%	40	50.0%
	4	50	53.2%	0	0.0%	0	0.0%	0	0.0%
	5	0	0.0%	26	25.0%	0	0.0%	26	32.5%
	6	28	29.8%	10	9.6%	6	7.0%	4	5.0%
	7	0	0.0%	0	0.0%	48	55.8%	0	0.0%
	Combined	94	100.0%	104	100.0%	86	100.0%	80	100.0%

Source: Author’s analysis.

**Figure 1.**
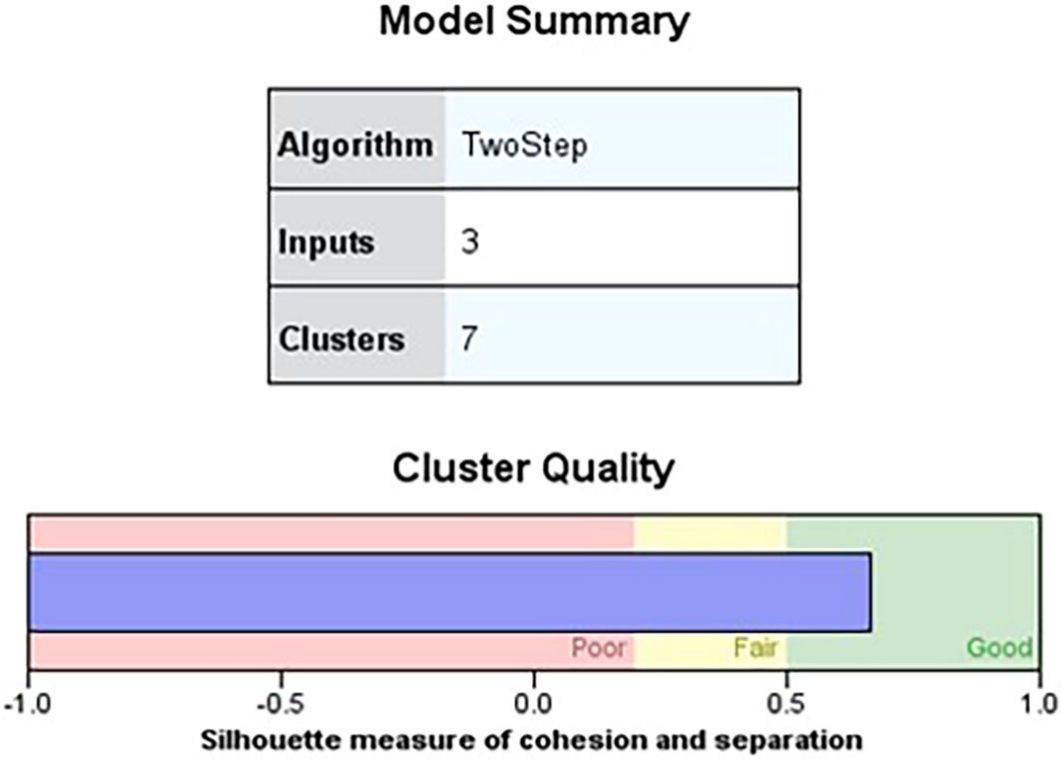
Model summary. Source: Author’s analysis.

In the 2-step cluster analysis, Table 7 displays the categorical variable and its corresponding prediction relevance, categorized into seven clusters, each with a unique pattern within the data. Figure 1 shows the quality of the cluster analysis based on three independent variables, indicating a favorable silhouette measure of cohesiveness and separation above the 0.7 threshold for good quality. Furthermore, the silhouette measure of cohesiveness and separation exceeding 0.5 suggests a favorable zone for cluster quality ( Cai et al., 2024; Hennig et al., 2015). The analysis revealed considerable differences among the independent factors, including the type of agricultural equipment used, irrigated land, and type of farming accessing individual clusters. However, the respondents in all groups primarily engaged in jasmine cultivation.

Table 1 outlines the order of input variables based on their significance within each cluster. In Figure 3 and Figure 4, Clusters One, Four, and Seven significantly impact irrigated land, whereas Clusters Two, Three, Five, and Six deem the impact of irrigated areas insignificant. Clusters Three and Five were distinguished by their unique cultivation practices, including the use of specific agricultural equipment and the adoption of different farming methods. Conversely, Clusters Two and Six exhibit statistical significance primarily regarding the farming methods employed, with other factors, such as irrigated area and agriculture equipment type, demonstrating less importance.

**Figure 2.**
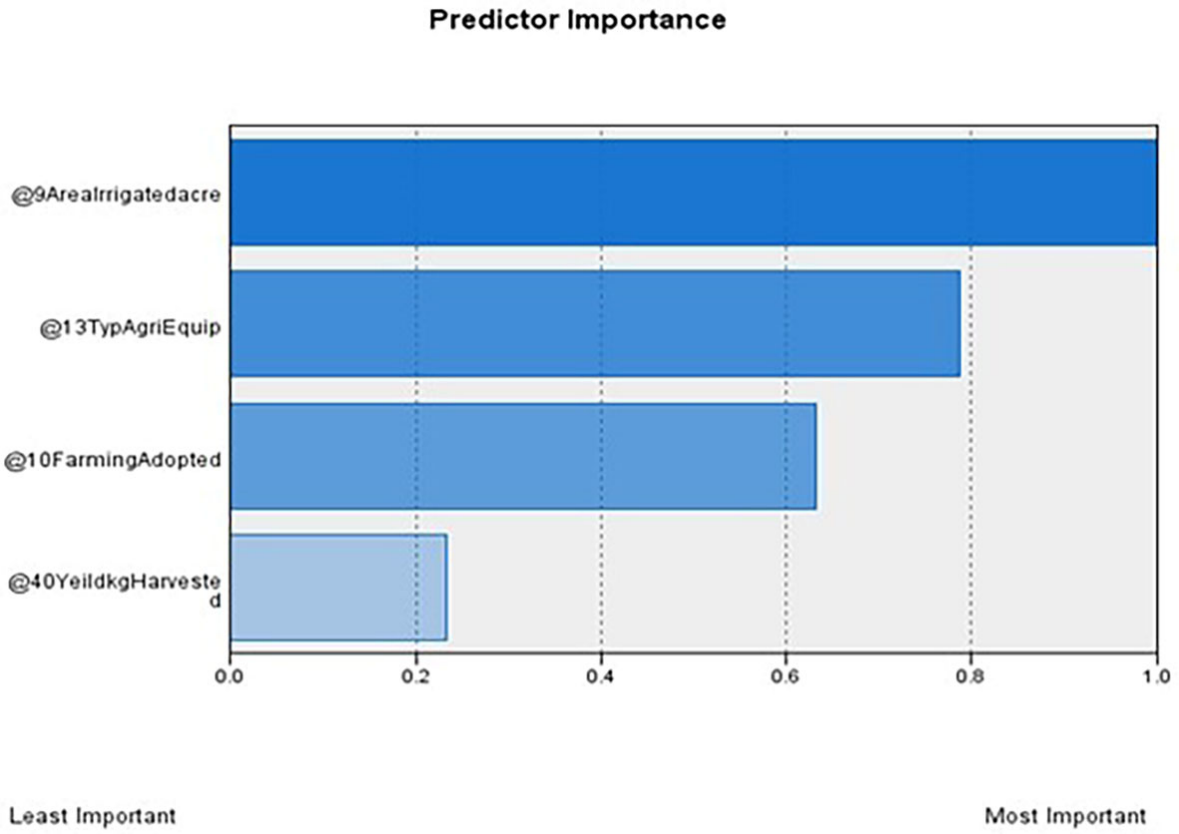
Predictor importance. Source: Author’s analysis.

**Figure 3.**
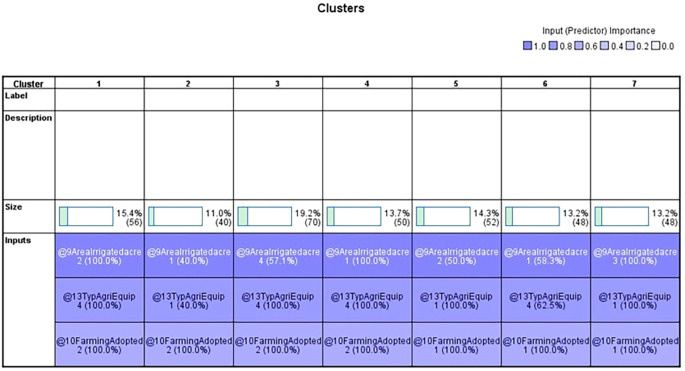
Categorical variables. Source: Author’s analysis.

**Figure 4.**
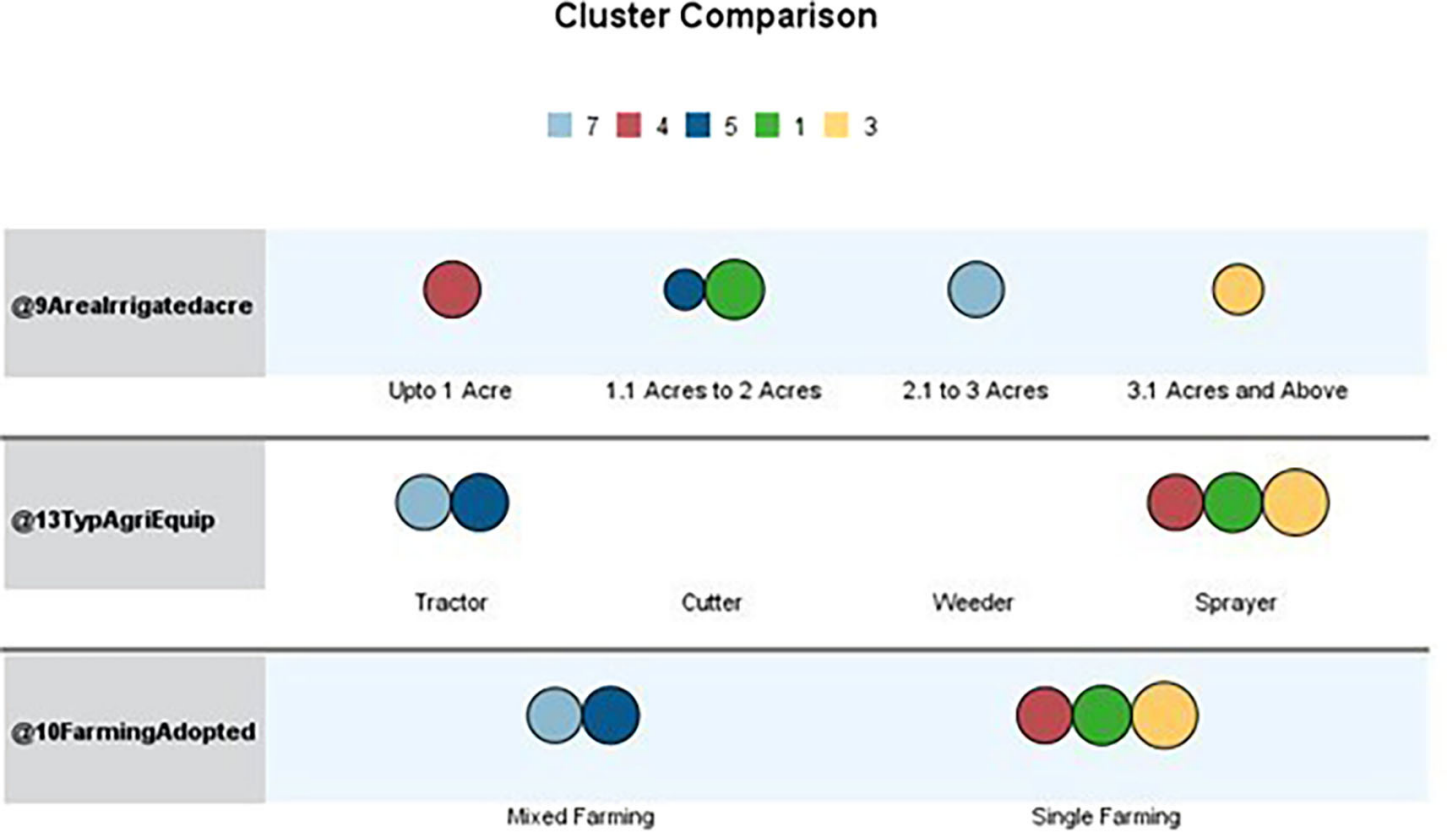
Cluster comparison. Source: Author’s analysis.

Overall, the analysis offers valuable insights into the distinct patterns of jasmine cultivation practices observed across various clusters, highlighting the significance of different input variables in shaping the patterns.

The data table shows the various farming practices and agricultural equipment usage across the clusters. Upon closer examination, it becomes apparent that three clusters engage in mixed cropping, whereas the others primarily practice monoculture farming. The sample includes individuals who practice both mixed and monoculture farming and those who exclusively practice monoculture farming.

Regarding agricultural equipment, clusters two, five, six, and seven predominantly use tractors. Cluster Six stands out for its diverse use of farming equipment. In contrast, clusters one, three, and four are characterised by farmers’ exclusive use of sprayers. Most farmers rely on sprayers for their agricultural activities.

Upon analyzing the clusters of irrigated areas, we discovered distinct patterns among farmers based on the size of their holdings. Farmers in the first cluster generally have between 1.1 and two acres of irrigated land. The second cluster includes those with holdings ranging from less than one acre to 3.1 acres or more, all under irrigation. Cluster three consists of farmers with 3.1 acres or more under irrigation. The fourth cluster comprises farmers in clusters five and six, with irrigated land ranging from 1.1 to 2 acres and 3.1 acres and above. The sixth cluster’s farmers have irrigated land ranging from less than 1 acre to 3.1 acres or more, with most concentrated in the 1.1- to 2-acre range. Finally, the seventh cluster comprised farmers with irrigated land between 2.1 and 3 acres. It is worth noting that most farmers tend to have between 1.1 and 2 acres of irrigated land.

After analyzing the characteristics of the surveyed farmers, we identified several distinct patterns. Cluster one (green) comprises farmers with irrigated land holdings between 1.1 and 2 acres who mainly practice single-crop farming and rely on sprayers for crop management. This cluster has the highest number of farmers in this land size range. Cluster three (Yellow) includes farmers with irrigated land of 3.1 acres or more who prefer a single-crop farming strategy and utilize sprayers as their primary farming equipment. Cluster four (Red) consists of farmers with smaller irrigated land holdings, up to one acre, who adopt a single-crop approach and favor sprayers.

Similar agricultural practices were demonstrated by Clusters Five (Royal Blue) and Seven (Sky Blue), which used only mixed farming and mostly tractors as their primary equipment. Nonetheless, Cluster Seven and Cluster Five have different land holdings: Cluster Seven owns 2.1 to 3 acres of irrigated land, whereas Cluster Five has 1.1 to 2 acres. Overall, it is clear that the clusters that were discovered differ in farming practices and agricultural equipment. Notably, none of the farming practices in these clusters involved mowers or weeders. The variations in land size, crop selection, and equipment preferences among the clusters underscore the heterogeneity of the agricultural community under study. Socioeconomically, they represent resource-constrained smallholders, many of whom own fewer than two acres and rely heavily on borrowed funds. Policy strategies, such as access to low-cost credit via cooperatives or SHGS, subsidized mechanization packages, and value addition, should be prioritized. This cluster reflects a transitional former segment that integrates traditional practices with modern technologies. Strategies to promote agri-entrepreneurship and FPO formation are required. A linkage between farmers and horticulture exports under the national scheme is required. It is also necessary to improve the post-harvest infrastructure and provide cold chain logistics to farmers.

## 5. Discussions

In the constantly changing field of agriculture, particularly Huvina Hadagali, the combination of tradition and innovation offers unique potential and challenges. The local government must uphold this delicate balance and be the guardian of the land and its agricultural heritage. While welcoming the winds of change blowing across industry, preserving the fabric of farming methods is imperative.

Initiatives to educate farmers about the advantages of contemporary farming methods are central to this endeavor. Farmers can obtain valuable insights into the potential benefits of implementing sophisticated technologies and optimizing irrigation methods through focused educational campaigns and outreach programs. Local governments can empower farmers to make well-informed decisions regarding their livelihood by eliminating impediments to modernization, such as high investment costs and operational complexity.

Financial support is another important factor for easing this shift in sustainability ( Prasetyo et al., 2020). By facilitating access to reasonably priced finance and offering incentives for adopting contemporary machinery and technologies, local governments may lessen financial strain on farmers and encourage investment in innovation. Furthermore, farmers can pool their resources and benefit from economies of scale using cooperative farming methods and collective negotiating power, thereby increasing their market competitiveness. However, it is important to remember the inherent value of traditions in the middle of the drive for advancement. India now presents a range of Central and State schemes specifically for GI-tagged products. The DPIIT offers a 100% grant of up to ₹1 crore for domestic and international fairs, packaging enhancements, and consumer awareness initiatives. The PM-FME provides a 35-50% subsidy on plant and machinery, along with a 90% grant for common facility centers within GI clusters. Organizations like APEDA, EPCH, and state facilitation cells support stalls, bar-coding, traceability, and legal assistance, while RKVY-RAFTAAR allocates up to ₹25 crore per cluster for integrated infrastructure. NABARD and ODOP contribute to FPO promotion and working-capital support, ensuring that finance and market access are readily available to registered GI producer groups in India. Deeply ingrained in Huvina Hadagali’s cultural legacy, the production of jasmine reminds us of the unbreakable bond between the land and its people. Local government bodies may ensure that farmers maintain the spirit of tradition by encouraging a sense of pride and continuity through the preservation and celebration of these traditional traditions.

## 6. Conclusion

It is of utmost importance to understand and support farmers who serve society in various ways. The region of our study is recognized by the produce of jasmine, and hence, farmers feel proud about their livelihood. However, time and preferences are changing, leading to many socioeconomic dimensions related to jasmine cultivation experiencing turbulence. Inexperienced farmers, low yield, lack of mechanization, and uncertainty in their income generation are typically the features of the farming community in Hadagali.

Rebuilding a lost reputation and injecting new blood into this holy work of floriculture requires many government interventions. Even though policies exist, farmers are unable to reap the benefits due to the political willpower of representatives and, thus, the government is unable to reach the needy. Academicians’ work needs to be geared up with field-based studies, giving some moral strength to the community at large. There is also a scope for the researcher to understand the changes in the Hadagali jasmine growing community with focusing on the ground root discussion.

### Ethics and consent statement

This research was conducted in strict accordance with existing ethical criteria owing to its involvement in human interactions. It has obtained the necessary ethical clearance (Institutional Ethics Committee (IEC) at Kasturba Medical College and Kasturba Hospital, Manipal, Karnataka, India- issued on 14 ^th^ December 2021, indicating that “project comes seems to come under pure economic, social and managerial research. If there are no health-related aspects involved, then you may proceed without IEC clearance” from the Institutional Ethics Committee (IEC) at Kasturba Medical College and Kasturba Hospital, Manipal, Karnataka, India. In addition, during the implementation of this non-experimental study, the researcher collected written informed consent from all participants, assuring them that their personal information would be handled with the highest level of confidentiality.

## Data Availability

The Data sheet is available in the DRYAD data repository. The details of the data sheet are given below. Repository Name: DRYAD - https://doi.org/10.5061/dryad.tb2rbp09d ( Joisa *et al*., 2024) The project contains the following underline data:
• Data File 1. Excel (364 coded respondents data sheets) Repository Name: DRYAD - https://doi.org/10.5061/dryad.tb2rbp09d ( Joisa *et al*., 2024) The project contains the following extended data:
• Questionnaire included in the Readme file. Data are available under the terms of the Creative Commons Zero “No rights reserved” data waiver (CC0 1.0 Public domain dedication).

## Software availability statement

IBM (SPSS) Statistics version 27 is a subscribed software for the Manipal Academy for Higher Education.
