# Supplementary material for: National Sentiments and Regional Flavour- A Socio-economic Study of Huvina Hadagali Jasmine
Source: F1000Res. 2025 Dec 22;13:1090. Originally published 2024 Sep 24. [Version 3] doi: 10.12688/f1000research.153101.3 (PMC12780521; doi:10.12688/f1000research.153101.3)
Supplement: Peer review history

# Reviewer report: Reviewer response for version 3

**DOI:** 10.5256/f1000research.192255.r427875

**Published:** 2026-01-07

**Authors:** B Suresh Lal

**Affiliations:** 1 Kakatiya University, Hanamkonda, India

**Introduction**

 The article offers a comprehensive introduction that highlights the importance of the research. It engages readers and enhances credibility. For example, it opens with a compelling statistic showing the economic impact of jasmine production: a rise in global floral trade revenues.

**Reviews**

 Building on these foundations, earlier studies, such as Patel (2015) and Chen (2017), have examined various aspects of jasmine cultivation and identified key research gaps. Patel's work highlighted the importance of sustainable farming practices, while Chen focused on technological advancements in farming techniques. However, a significant gap exists in understanding the socio-economic impacts of these practices on the local communities involved. Identifying this gap underscores the need for further research, thereby enhancing scholarly critique.

**Research Design**

 With meticulous attention to detail, the article's research methods are skillfully devised and executed, showcasing a profound understanding of the subject. The methodology provides a comprehensive description of the procedures and tools employed, thereby ensuring validity and reliability. This study used purposive sampling to select participants actively involved in jasmine cultivation in the Huvina Hadagali region, and the two-step clustering algorithm utilises the Bayesian information criterion (BIC) to determine the optimal number of clusters automatically. Potential methodological limitations include the inherent biases of purposive sampling, which may not fully reflect the diversity within the broader population of jasmine cultivators. Additionally, reliance on the BIC in cluster determination, while generally effective, may not account for all variables that affect optimality.

**Findings and Discussion**

 The study provides clear demographic information and cluster analysis for jasmine cultivators. Differences in land size, crop choices, and equipment preferences highlight the agricultural community's diversity. Socioeconomically, these growers are resource-constrained smallholders. Many own less than two acres and often rely on borrowed funds.

**Conclusion**

 In conclusion, the article's ending clearly shows the author's grasp of the subject. It significantly contributes to the field by addressing gaps in the literature on the socio-economic impacts of sustainable and advanced jasmine farming techniques. The research emphasises the diversity among jasmine farmers and the varied economic implications of their agricultural practices. By calling for further exploration of these socio-economic effects, the article strengthens the discourse and paves the way for future studies on agrarian sustainability.

Is the work clearly and accurately presented and does it cite the current literature?

Yes

If applicable, is the statistical analysis and its interpretation appropriate?

Yes

Are all the source data underlying the results available to ensure full reproducibility?

Yes

Is the study design appropriate and is the work technically sound?

Yes

Are the conclusions drawn adequately supported by the results?

Yes

Are sufficient details of methods and analysis provided to allow replication by others?

Yes

Reviewer Expertise:

Development economics, Health economics, and the Economics of Tribal Studies.

I confirm that I have read this submission and believe that I have an appropriate level of expertise to confirm that it is of an acceptable scientific standard.

# Reviewer report: Reviewer response for version 2

**DOI:** 10.5256/f1000research.181675.r427876

**Published:** 2025-11-22

**Authors:** Dr Ajay Massand

**Affiliations:** 1 PES University, Bangalore, India

**Summary**

 The research paper presents the socio-economic status of the Jasmine flower growers in Vijayanagar district in Karnataka. The study has large sample of 364 jasmine flower growers' data collected through systematic questionnaire. The study has used purposive data sampling. It analyzed the data using frequency analysis and two-step cluster analysis. 

**Introduction**

 The study attempts to motivate the selection of specific topic on Jasmine flower Huvina Hadagali of Karnataka, It has been cited well and written constructively.  

**Need for Improvement- **Researcher should add the total market for jasmine flower in India. how much is the contribution of Huvina Hadagali production to the total market, how many families/households are involved in this production, When the area got GI tag (recently?) etc. This will enhence the support to the selection of the topic for the study.

**Literature Review**

 It is comprehensive with latest literature. 

**Study Design**

 The study used purposive sampling with 364 jasmine cultivators. It is a good sample for data collection. Even the pilot study is also conducted. 

**Need for Improvement-** *I suggest adding mixed method by conducting interviews with some of the respondents that highlight the improvement in socio economic status over the years. Do record it.*

**Findings and discussion**

 The study provides demographical, socio-economic factors along with the challenges faced by the farmers. It also distingue the characteristics of different clusters. 

***Need for improvement- **It should clearly highlight the socio-economic well-being of farmers. You may collect more information on income improvement, government schemes and subsidies if any, growth in production, export quantity etc.*

**Discussion**

 It discusses about government support and financial support. However, author should discuss the schemes related to Jasmine flower by different states and central govt. scheme. Why it was under the GI tag and related to its export's promotion. Also, analyze and mention how commercial banks can extend their help for loan accessibility. 

**Conclusion**

*It is provided in general.*

***Need for improvement- **what exactly you found about the socio-economic status of the farmers, how govt, can intervene, why your results are limited to a particular area that mislead the overall results so need to perform an extensive study covering all the area's growing Jasmine in India*

Is the work clearly and accurately presented and does it cite the current literature?

Yes

If applicable, is the statistical analysis and its interpretation appropriate?

Yes

Are all the source data underlying the results available to ensure full reproducibility?

Yes

Is the study design appropriate and is the work technically sound?

Partly

Are the conclusions drawn adequately supported by the results?

Partly

Are sufficient details of methods and analysis provided to allow replication by others?

Yes

Reviewer Expertise:

Financial Economics, Macroeconomics, Research Methodology

I confirm that I have read this submission and believe that I have an appropriate level of expertise to confirm that it is of an acceptable scientific standard, however I have significant reservations, as outlined above.

# Response

**Published:** 2025-11-25

**Authors:** Harisha G Joshi

**Affiliations:** Department of Commerce, Manipal Academy of Higher Education, Manipal, Karnataka, India

Greetings, 

 Dear  Reviewer, 

 Thank you for reviewing our article and providing insightful suggestions to improve its standards. The said suggestions have been incorporated in the revised manuscript wherever possible. Kindly do the needful.

# Reviewer report: Reviewer response for version 1

**DOI:** 10.5256/f1000research.167949.r374599

**Published:** 2025-05-02

**Authors:** Rajib Sarkar

**Affiliations:** 1 Adamas University, Kolkata, West Bengal, India

**1. Summary**

 The paper presents a socio-economic analysis of jasmine cultivation in Huvina Hadagali, Karnataka, India. Using data from 364 jasmine growers, the study explores the economic contribution of jasmine cultivation to rural livelihoods and identifies the challenges faced by farmers. The methodology involves frequency analysis and two-step clustering through SPSS software.

**2. Novelty and Significance**

 •    Strengths:

 o    The topic is regionally significant and underexplored, especially concerning the socio-economic dimensions of GI-tagged floriculture products like Hadagali Jasmine.

 o    The study captures the socio-economic fabric of a traditional and culturally significant agricultural practice.

 •    Weaknesses:

 o    The paper's contribution to wider academic knowledge is moderate. Although the context is important regionally, broader theoretical contributions (e.g., linking findings to global floriculture market trends) are missing.

**3. Clarity and Structure**

 •    Strengths:

 o    Clear and structured writing.

 o    Logical progression from background to methods, results, and discussion.

 o    Data tables are well-organized and supportive of the narrative.

 •    Weaknesses:

 o    Language could be polished further to avoid minor grammatical and phrasing issues.

 o    Some sections (e.g., Introduction) are slightly repetitive regarding jasmine's cultural importance.

 o    The abstract could briefly highlight key recommendations rather than ending only on challenges.

**4. Methodology**

 •    Strengths:

 o    The use of a structured questionnaire and appropriate statistical techniques (frequency analysis, two-step clustering) is suitable for the objectives.

 o    Good sample size (n=364) relative to the region under study.

 •    Weaknesses:

 o    More justification for using purposive sampling over random sampling would strengthen the research design.

 o    There is limited discussion on questionnaire validation or reliability testing (e.g., pilot study, Cronbach's alpha).

 o    Two-step clustering is appropriately used, but the rationale for cluster number selection (automatic vs. manual) could be more clearly explained.

**5. Analysis and Interpretation**

 •    Strengths:

 o    The demographic, economic, labour, and market analyses are comprehensive.

 o    Cluster analysis effectively distinguishes different farming practices.

 •    Weaknesses:

 o    Discussion of the cluster results lacks depth: how do these clusters inform targeted policy measures?

 o    Discussion could better connect findings to existing literature (both national and global) to highlight similarities or contrasts.

**6. Conclusion and Policy Implications**

 •    Strengths:

 o    Clear recognition of the need for policy interventions, financial support, and modernization.

 •    Weaknesses:

 o    Policy recommendations remain general; specific actionable strategies (e.g., models for cooperative development, suggestions for post-harvest technology deployment) would enhance practical relevance.

**7. Ethical and Data Transparency**

 •    Strengths:

 o    Ethical considerations are well-addressed, including informed consent.

 o    Data availability on Dryad enhances transparency and reproducibility.

**8. Typographical and Formatting Issues**

**•    Minor issues found:**

 o    "Nevertheless, the highlighted obstacles..." (Abstract) – sentence structure could be polished.

 o    "The local government must uphold this delicate balance..." (Discussion) – overly editorial in tone; can be more neutral.

 o    Use of "research highlights" multiple times could be varied stylistically.

**•    Figures:**

 o    Some figure legends (e.g., Figure 1, Figure 2) are too brief. Expand to make figures self-explanatory.

**9. Specific Suggestions for Improvement**

 •    Clarify rationale for purposive sampling and how representativeness was ensured.

 •    Improve consistency in language (minor grammatical edits suggested).

 •    Expand the discussion of the two-step cluster findings with more linkage to socio-economic strategies.

 •    Suggest specific policy interventions (e.g., model cooperatives, farmer credit facilities, transport infrastructure improvements).

 •    Improve figure captions for clarity.

Is the work clearly and accurately presented and does it cite the current literature?

Yes

If applicable, is the statistical analysis and its interpretation appropriate?

Partly

Are all the source data underlying the results available to ensure full reproducibility?

Yes

Is the study design appropriate and is the work technically sound?

Yes

Are the conclusions drawn adequately supported by the results?

Yes

Are sufficient details of methods and analysis provided to allow replication by others?

Partly

Reviewer Expertise:

My research interest encompasses on data driven policy decision, sustainability, application of remote sensing and GIS for environmental management.

I confirm that I have read this submission and believe that I have an appropriate level of expertise to confirm that it is of an acceptable scientific standard, however I have significant reservations, as outlined above.

# Response

**Published:** 2025-05-22

**Authors:** Harisha G Joshi

**Affiliations:** Department of Commerce, Manipal Academy of Higher Education, Manipal, Karnataka, India

Dear Reviewer,

 We sincerely thank you for your thoughtful and constructive feedback on our manuscript. Your insightful suggestions have been invaluable in enhancing the overall quality and clarity of our work.

 We have carefully reviewed and addressed each of your comments in detail. All suggested revisions have been incorporated into the revised manuscript, including improvements to the methodology section, data interpretation, and literature citations. These changes have strengthened the manuscript and helped us present our research more effectively.

 We are grateful for your time, effort, and expertise in reviewing our work, and we truly appreciate your contribution to improving the quality of our research.

 Thank you once again for your support and guidance.
